# WNT3 Inhibits Cerebellar Granule Neuron Progenitor Proliferation and Medulloblastoma Formation via MAPK Activation

**DOI:** 10.1371/journal.pone.0081769

**Published:** 2013-11-26

**Authors:** Sandrine L. Anne, Eve-Ellen Govek, Olivier Ayrault, Jee Hae Kim, Xiaodong Zhu, David A. Murphy, Linda Van Aelst, Martine F. Roussel, Mary E. Hatten

**Affiliations:** 1 Laboratory of Developmental Neurobiology, The Rockefeller University, New York, New York, United States of America; 2 Department of Tumor Cell Biology, St. Jude Children’s Research Hospital, Memphis, Tennessee, United States of America; 3 Cold Spring Harbor Laboratory, Cold Spring Harbor, New York, United States of America; University of Nebraska Medical Center, United States of America

## Abstract

During normal cerebellar development, the remarkable expansion of granule cell progenitors (GCPs) generates a population of granule neurons that outnumbers the total neuronal population of the cerebral cortex, and provides a model for identifying signaling pathways that may be defective in medulloblastoma. While many studies focus on identifying pathways that promote growth of GCPs, a critical unanswered question concerns the identification of signaling pathways that block mitogenic stimulation and induce early steps in differentiation. Here we identify WNT3 as a novel suppressor of GCP proliferation during cerebellar development and an inhibitor of medulloblastoma growth in mice. WNT3, produced in early postnatal cerebellum, inhibits GCP proliferation by down-regulating pro-proliferative target genes of the mitogen Sonic Hedgehog (SHH) and the bHLH transcription factor *Atoh1*. WNT3 suppresses GCP growth through a non-canonical Wnt signaling pathway, activating prototypic mitogen-activated protein kinases (MAPKs), the Ras-dependent extracellular-signal-regulated kinases 1/2 (ERK1/2) and ERK5, instead of the classical β-catenin pathway. Inhibition of MAPK activity using a MAPK kinase (MEK) inhibitor reversed the inhibitory effect of WNT3 on GCP proliferation. Importantly, WNT3 inhibits proliferation of medulloblastoma tumor growth in mouse models by a similar mechanism. Thus, the present study suggests a novel role for WNT3 as a regulator of neurogenesis and repressor of neural tumors.

## Introduction

During normal cerebellar development, the extraordinary amplification of granule cell progenitors (GCPs) generates vast numbers of granule neurons, up to 45 billion in the human cerebellum. This unique postnatal expansion of neuronal precursors is driven in large part by the mitogen Sonic Hedgehog (SHH) [1], and deregulation of the SHH signaling pathway in GCPs gives rise to a subset of medulloblastoma [2,3,4,5,6,7,8,9,10,11,12,13,14,15,16,17,18,19,20,21,22,23,24], which is the most prevalent metastatic brain tumor of childhood [5,8]. Thus, GCP proliferation provides a model for normal CNS neurogenesis and for identifying signaling pathways pertinent to this devastating disease [8]. To date, studies on GCP proliferation have mainly focused on SHH and its receptor, Patched (PTCH1) [1,24]; JAG1 and its receptor, NOTCH2, [25]; Trp53 and the cyclin-dependent kinase (CDK) inhibitor p18^Ink4^/Cdkn2c [26]; and Mycn and the D-type cyclins [27,28]. However, a critical unanswered question concerns the identification of signaling pathways that block mitogenic stimulation and induce early steps in differentiation, and as such may offer insight into potential treatments for medulloblastoma. 

Initially described in *Drosophila*, murine WNT proteins are a large family (>19) of highly conserved secreted signaling molecules that regulate cell-cell interactions during the development of multiple organ systems, including the CNS. WNT proteins regulate diverse cellular processes, including proliferation, cell fate determination, differentiation, axon growth and synapse formation [29]. With regard to cerebellar development, *Wnt1* is required to induce the isthmus organizer, which establishes the midbrain/hindbrain boundary to specify the cerebellar territory [30,31,32,33]. Deregulation of WNT signaling components in early Nestin-expressing progenitors and GCPs impairs proliferation and causes premature differentiation, resulting in cerebellar abnormalities [34,35,36]. At later stages of cerebellar development, WNT7a functions in the terminal differentiation of cerebellar granule neurons, remodeling axons and growth cones of mossy fiber afferents as they form synaptic connections with granule neuron dendrites [37,38,39,40]. Although aberrant WNT signaling affects proliferation and has been implicated in medulloblastoma [3,4,5,9,41,42,43,44,45,46,47,48,49,50], this subset of medulloblastomas is thought to arise outside of the cerebellum from progenitors in the dorsal brainstem [45].

Most studies have focused on the role of canonical, β-catenin-dependent, WNT signaling in development. Canonical WNT signaling stabilizes β-catenin, which forms complexes with TCF/LEF transcription factors and controls the expression of many genes important for CNS neurogenesis, including *Mycn*, *cyclin D1* (*Ccnd1*), *neurotrophin 3* (*Ntf3*), *connexin-43* (*Gja1*) and metalloproteases (*Mmps*) [29,51,52]. Non-canonical WNT signaling, which functions in a β-catenin-independent manner, is less well understood. Non-canonical pathways include WNT/PCP (planar cell polarity) signaling, the WNT/Ca ^2+^ pathway and the WNT/Jun N-terminal kinase (JNK) pathway [53,54,55,56,57,58]. While some WNTs seem to preferentially signal through a canonical or non-canonical pathway, the activity of WNTs and their effects on cell growth and development depend on the cellular context and the receptors involved [59]. Although studies have identified a role for downstream signaling components of canonical WNT signaling in early GCP development, which particular WNTs regulate postnatal GCP proliferation and differentiation, as well as the signaling pathways they utilize, remain to be defined.

Here we examined the role of WNT3 in GCP proliferation and medulloblastoma growth. We provide evidence that WNT3 inhibits GCP proliferation *in vitro* and *ex vivo*. WNT3 attenuates GCP growth by inhibiting pro-proliferative SHH-induced transcriptional responses and the bHLH transcription factor *Atoh1* [60]. WNT3 acts through a non-canonical WNT signaling pathway that involves phosphorylation of prototypic mitogen-activated protein kinases (MAPKs), the Ras-dependent extracellular-signal-regulated kinases 1/2 (ERK1/2) and ERK5. Importantly, WNT3 inhibits proliferation of medulloblastoma tumor growth in mouse models by a similar mechanism. Thus, WNT3 is a novel growth suppressor during cerebellar development and an important regulator of medulloblastoma formation.

## Materials and Methods

### Ethics statement

All animal work was performed as required by the United States Animal Welfare Act and the National Institutes of Health's policy to ensure proper care and use of laboratory animals for research. Mice were housed in accredited facilities of the Association for Assessment of Laboratory Animal Care (AALAC) in accordance with the National Institutes of Health guidelines.

All animal work conducted to obtain primary GCPs was performed under established guidelines and supervision by the Institutional Animal Care and Use Committee (IACUC) of The Rockefeller University under protocol number 1009. All efforts were made to minimize suffering. Euthanasia of neonatal mouse pups was carried out by reducing their core body temperature, by placing them in the refrigerator (4°C) for approximately ten minutes, followed by decapitation. Chilling early postnatal mouse pups is known to be a painless and fully reversible method for inducing "anesthesia", and is preferable to the use of anesthetic overdose in neonates because it is painless and avoids any compromise to the experiments that drugs might cause. 

 All animal work conducted to obtain primary medulloblastoma cells was performed under established guidelines and supervision by the St. Jude Children's Research Hospital's Institutional Animal Care and Use Committee (IACUC) permit number A3077-01, that approved all procedures in this study as required by the United States Animal Welfare Act and the National Institutes of Health's policy to ensure proper care and use of laboratory animals for research under the protocol 378. All surgery was performed under sodium pentobarbital anesthesia, and all efforts were made to minimize suffering, including the administration of analgesics when necessary. Mice were monitored daily because we knew that the animals would get tumors, and mice that developed medulloblastoma tumors were humanely euthanized using CO_2_ once we detected signs of sickness, including failure to thrive, head dome, and gait problems.

### GCPs culture

Granule cell progenitors (GCPs) were purified from P6 C57Bl6/J mice (Jackson Laboratory, Bar Harbor, ME), by Percoll gradient sedimentation and pre-plating, as described previously [61]. The purity of GCPs was greater than 98%. Briefly, purified GCPs were re-suspended in serum-free medium (DMEM/F12, 1.5% Glucose, 0.1% Na bicarbonate, 5 mM Hepes, 20 mM Glutamine, 1% N2). In some experiments, cells were grown on Poly-D-Lysine (PDL, 10 µg/ml, Sigma), MATRIGEL (Beckton Dickson, Growth Factor Reduced, 0.2 mg/ml) or laminin (10 µg/ml, Invitrogen) coated dishes. For immunoblotting analysis, 3.75x10^6^ GCPs/ml in serum-free media were plated in 6 well dishes and cultured for 24 h. Cells were treated with WNT3 conditioned medium (100 ng/ml), commercially available WNT3 (250 ng/ml, Creative Biomart), BMP7 (200 ng/ml, R&D), SHH (3 µg/ml, R&D), 6-bromoindirubin-3’-oxime (BIO, 5 µM), Cyclohexamide (10 µg/ml, Sigma), PD98059 (50 µM, Cell Signaling), CHIR99021 (3 µM, StemGent) or LiCl (10 nM) for 24 h, unless otherwise noted. Afterwards, GCPs were lysed with TRIS buffer containing Triton X-100 (50 mM Tris-HCl pH 7.5, 100 mM NaCl, 50 mM NaF, 5 mM EDTA, 1% Triton X-100, 40 mM β glycerophosphate, 200 µM sodium orthovanadate, 100 mM PMSF and protease inhibitor cocktail (Roche). Western blots were performed a minimum of three times, and a representative image is shown. 

### Quantitative PCR

Quantitative PCR experiments were subjected to all “essential” qPCR publishing criteria based on the recommendations of the MIQE guidelines report [62]. All primers used for final experiments were tested on gradient PCR for annealing temperature and single product amplification. Primer efficiency, determined by standard curve using cDNA, was between 1.95 and 2.05. Melt curve analysis showed a single well-defined peak at the predicted melting temperature for each product. Both no template and reverse transcriptase negative controls showed no amplification. Primers are listed in Table S1.

**Figure 1:**
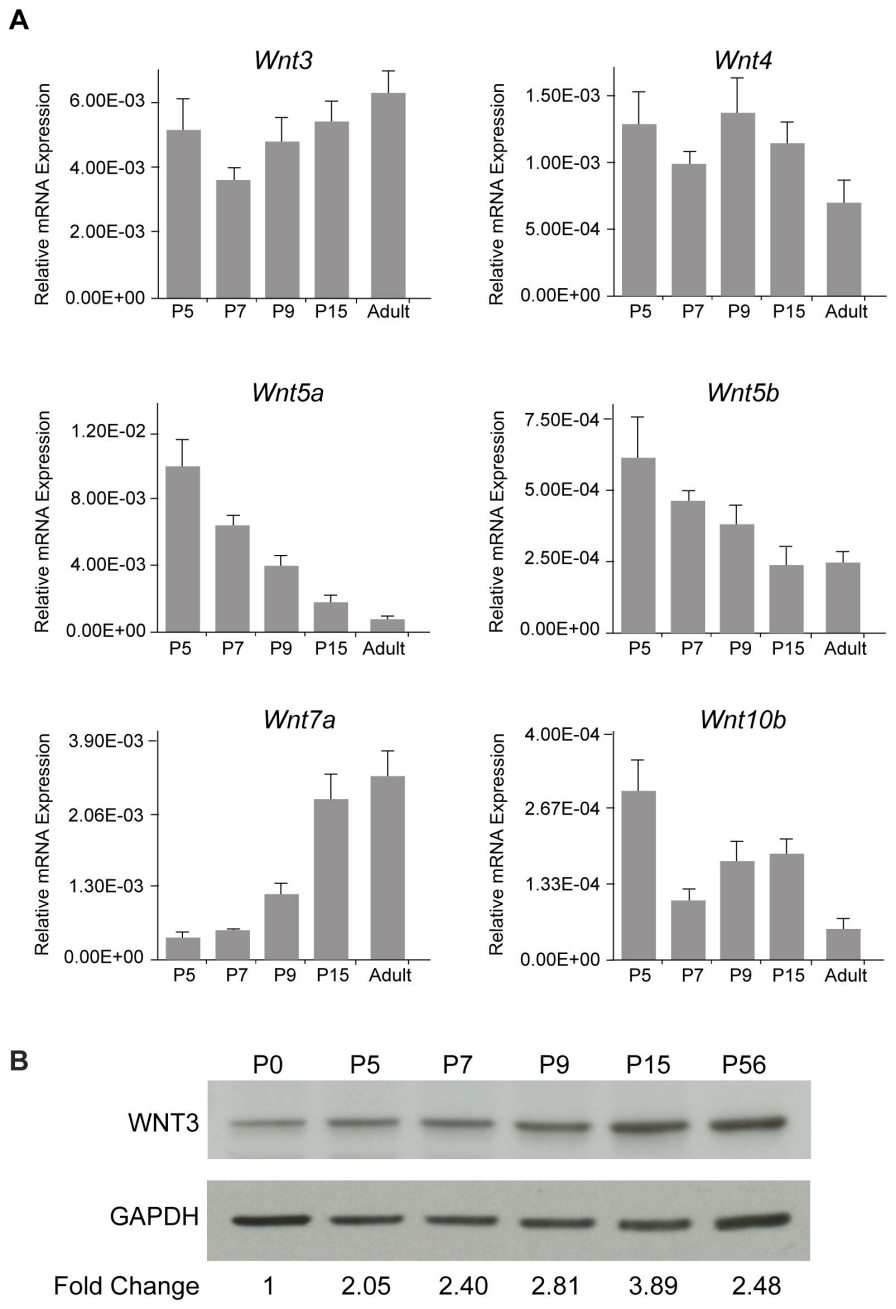
Gene expression profile of Wnts in cerebellar development. (*A*) qPCR of Wnt3, Wnt4, Wnt5a, Wnt5b, Wnt7a, and Wnt10b, in the mouse cerebellum from postnatal day 5 (P5) to adult. Wnt levels were normalized against β-2-microglobulin (B2M), hyporanthine-guanine phosphoribosyltransferase (HPRT1) and ribosomal subunit 18s (M18s). (*B*) WNT3 protein levels during cerebellar development. Immunoblotting of cerebellar lysates from P0 to P56 (adult) with an anti-WNT3 antibody showed an increase in WNT3 protein during cerebellar development.

For analysis of WNT and SHH signaling components in Figure 1A and Figure S5 for each developmental stage (P5 to adult; adult is defined as P55 or older), cerebellar tissue was removed, snap frozen with liquid nitrogen and stored at -80°C. RNA was extracted from the frozen tissue with a Teflon-glass homogenizer using TriZol reagent (Ambion). RNA quantity was assayed using a Nanodrop (Thermo Scientific). For each sample, an equal amount of RNA was digested with DNAse I (Invitrogen) and then concentrated and purified using a column purification kit (Qiagen RNeasy). Bioanalysis (Agilent 2100) showed negligible levels of ethanol, DNA or protein in the samples with RNA integrity (RIN) >9 for all samples used. RNA samples were then reverse transcribed to cDNA using random hexamers and oligo DT primers (Roche Transcriptor). We used a geometric averaging protocol to normalize gene expression against three control genes: beta-2-microglobulin (B2M), hyporanthine-guanine phosphoribosyltransferase (HPRT1) and ribosomal subunit 18s (M18s) [63,64]. Results were analyzed on a Roche LC 480 II system using Roche LC 480 software.

**Figure 2:**
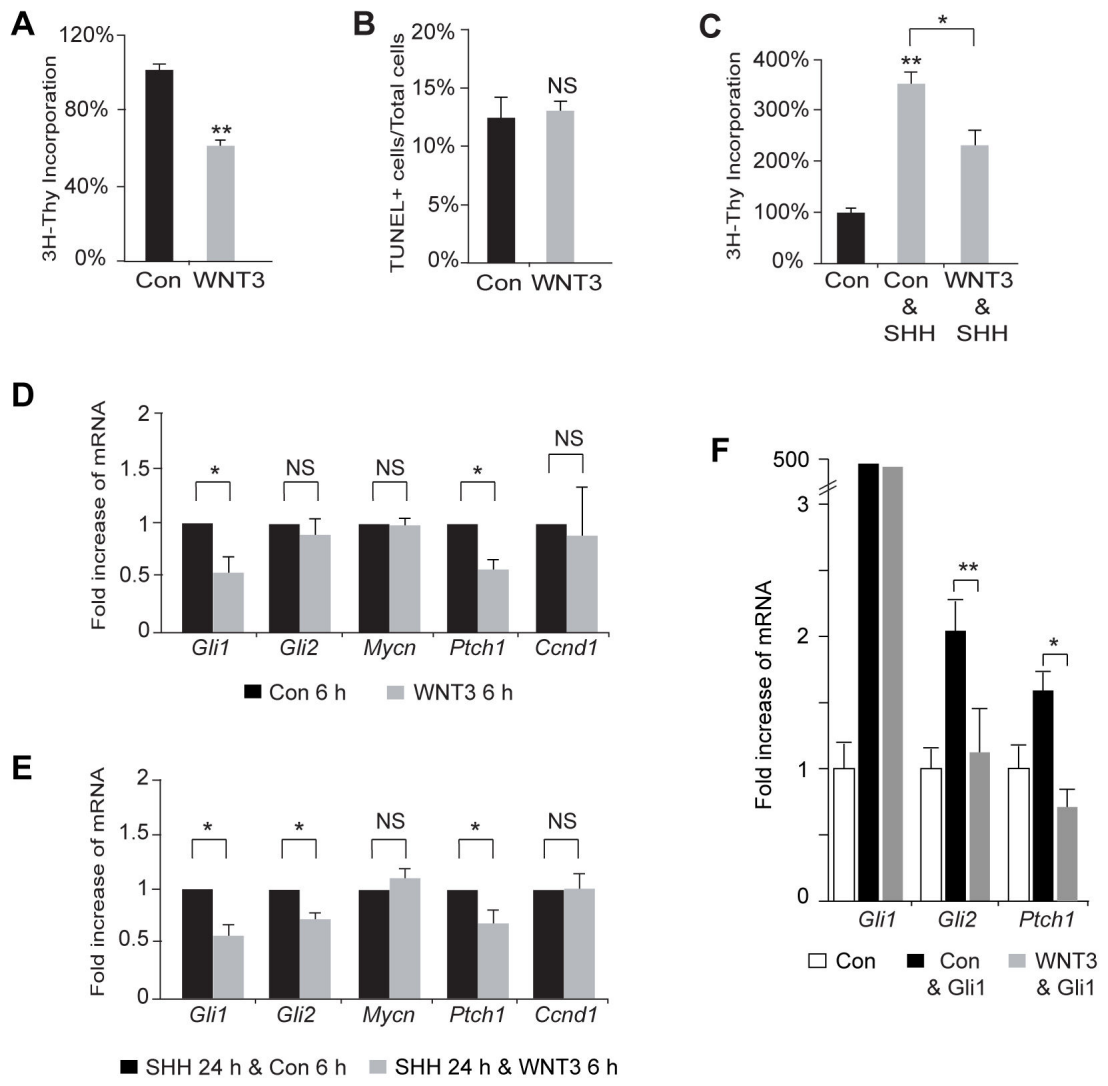
WNT3 decreases GCP proliferation by inhibiting SHH dependent gene transcription. (*A*) WNT3 decreased proliferation of GCPs by 40.3± 5.0% of the control (Con) (n=3) as measured by [^3^H]-Thymidine incorporation assay. (*B*) WNT3 did not increase cell death of GCPs in a TUNEL assay at 2 DIV (Con, 12.5±1.6%; WNT3, 13.1±0.7% (n=3)). (*C*) WNT3 decreased SHH dependent proliferation of GCPs (Con & SHH, 354.8±18.7%; WNT3 & SHH, 232.9±28.7% (n=3)). (*D*) WNT3 decreased Gli1, and *Ptch1* mRNA levels after 6 h of treatment (Con=1; WNT3: Gli1=0.56±0.10, Gli2=0.90±0.10, Mycn= 0.99±0.05, *Ptch1*=0.58±0.09, and *Ccnd1*=0.89±0.09). (*E*) Treatment of GCPs with WNT3 antagonized the transcription of SHH targets. SHH was added for 24 h and WNT3 was added for 6h. (SHH/Con=1; SHH/WNT3: Gli1=0.62±0.04, Gli2=0.75±0.02, Mycn=1.12±0.05, *Ptch1*=0.70±0.11 and *Ccnd1* =1.03±0.12.). (*F*) After 24 h of GLI1 overexpression in GCPs, *Gli2*, and *Ptch1* mRNA levels increase in the control condition (Gli1=464±119, Gli2=2.04±0.25, *Ptch1*=1.59±0.14) and this effect is inhibited in the presence of WNT3 (Gli1=314±166, Gli2=1.14±0.3, *Ptch1*=0.71±0.12). Data represent the mean ± s.e.m.: *p<0.05, **p<0.01, ***p<0.001. NS, not significant.

 For the analysis of SHH target genes in the presence and absence of WNT3 in Figure 2D, GCPs were treated with conditioned medium or WNT3 for 6 h before isolating RNA. For the analysis of SHH target genes in the presence and absence of WNT3 and SHH in Figure 2E, GCPs were treated with SHH for 24 h, and control medium or WNT3 was added during the last 6 h before isolating RNA. For the GLI1 overexpression experiment in Figure 2F, GCPs were electroporated with a control vector or pSR-Gli1 vector [65] and treated with control medium or WNT3 for 24 h. For the analysis of SHH target genes in the presence and absence of WNT3 and SHH in Figure 2D,E and the GLI1 overexpression experiment in Figure 2F, RNA was prepared using TRiZol reagent (Ambion). cDNA was synthesized using the SuperScript First-Strand Synthesis System, and qPCR assays were performed using iTaq SYBR Green Supermix with ROX (Bio-Rad). *β-actin* was used as an internal control. qPCR results for the experiments shown in Figures 2D,E and 2F were analyzed with a MyiQ single color real-time PCR detection system (Bio-Rad).

### Expression constructs, retrovirus and WNT3 production

Full-length mouse *Wnt3* cDNA was amplified from RNA prepared from P6 cerebellum using the following primers: (Forward/EcoRI) 5’-GGAATTCTCGGCGCTGCTTCTAATG-3’ and (Reverse/HpaI) 5’-GGTTAACCCCTTCCCAGTGCCCTGG-3’, and inserted into the *pCXi* expression vector by EcoRI/HpaI. We stably infected NIH3T3 cells with the retroviral vector *pCxi* containing *Wnt3* cDNA, cultured the cells 5 days in 0.5% calf serum, BME, and pen/strep and collected the medium. 

WNT3 protein is 39 kDa. To produce WNT3 conditioned medium, 400 ml of control or WNT3 supernatant were filtered using four 50 kDa MWCO PES Sartorius Vivacell 100 ml ultra centrifuge filters to remove proteins larger than 50 kDa. The eluate (approximately 360 ml total) was then concentrated using four 10 kDa MWCO PES Sartorius Vivacell 100 ml ultra centrifuge filters to a top fraction volume of 10 ml each (40 ml total) to remove proteins smaller than 10 kDa. The 40 ml of concentrated 10 kDa top fraction was then further concentrated using four 15 ml Amicon Ultra Ultracel 10K centrifugal filters, to a final volume of 1 ml per filter (4 ml total). The resulting 4 ml of 100x concentrated supernatant was dialyzed in Slide-A-Lyzer dialysis cassettes (Pierce, MW 7000) in BME medium at 4°C overnight. Contamination by other WNTs (WNT1, 3a, 5a, 7a) was excluded by immunoblotting control and WNT3 conditioned medium for these WNT proteins, and extensive tests were performed to rule out the presence of other growth factors (data not shown). WNT3 conditioned medium will therefore be referred to as “WNT3” and control conditioned medium referred to as “control”.

Virus production and infection using the *pMSCV-Atoh1-IRES-GFP* vector were performed as described previously [66].

### Antibodies

The following primary antibodies were used for either immunocytochemistry or Western blot analysis: anti-WNT3 (mouse, Invitrogen), anti-GFAP (mouse, Dako), anti-Calbindin (rabbit, Swant), anti-β-catenin (rabbit, Cell Signaling), anti-active-β-catenin (mouse, Upstate Biotechnology), anti-phospho-Y216-GSK3β and anti-GSK3β (mouse, BD Transduction), anti-MYCN (rabbit, C-19, Santa Cruz), anti-phospho-p38 (rabbit, Cell Signaling), anti-p38 (rabbit, Abcam), anti-phospho-JNK (mouse, Cell Signaling) and anti-JNK (rabbit, Cell Signaling), anti-phospho-ERK1/2 and anti-ERK1/2 (rabbit, Cell Signaling), anti-phospho-ERK5 and anti-ERK5 (rabbit, Cell Signaling), anti-Pax6 (rabbit, Covance) anti-phospho-AKT (rabbit, Cell Signaling), anti-Cyclin D1 (mouse, Santa Cruz) and anti-GAPDH (mouse, Chemicon), anti-phospho-Smad 1/5/8 (rabbit, Cell Signaling), anti-Smad1 (rabbit, Dr. J. Massague), and anti-β-actin (mouse, MP Biomedicals). Secondary antibodies used (Molecular Probes) included goat anti-rabbit IgG-Alexa 488 Fluor, goat anti-rabbit IgG-Alexa 555 Fluor, goat anti-mouse IgG-Alexa 488 Fluor, and goat anti-mouse IgG-Alexa 555 Fluor (all used at 1:500). DRAQ5 was used at 1/1000 (647 nm excitation, BioStatus).

### [^3^H]-Thymidine incorporation assay

To assay [^3^H]-Thymidine incorporation, we plated purified P6 cerebellar GCPs (300,000 cells per well) in 96-well dishes and treated them with WNT3, SHH or other proteins or inhibitors for 24 h. WNT3 was used at 100 ng/ml, SHH (R&D) at 3 µg/ml, BMP2 (R&D) at 150 ng/ml, BMP4 (R&D) at 100 ng/ml, BMP6 (R&D) at 150 ng/ml, BMP7 (R&D) at 200 ng/ml, Noggin (R&D) at 1 µg/ml and PD98059 (Cell Signaling) at 50 µM. 1 µCi of [methyl-^3^H]-thymidine (Amersham and MP Biomedicals, LLC) was added to each well after 1 day *in vitro* (DIV) and the cells were harvested and analyzed using TOP-Count (Perkin Elmer). 

### Terminal Deoxyuridine Nick End-Labeling (TUNEL)

To assay apoptosis, purified GCPs were plated at 2x10^6^ cells/cm^2^ in serum-free medium onto plates coated with PDL and MATRIGEL for 2 days, and TUNEL reaction was performed as described by the manufacturer. Confocal images of DRAQ5 and TUNEL stained cells were acquired with a Radiance 2000 confocal laser-scanning microscope (Bio-Rad), and labeled cells were counted using MetaMorph software (Image One).

### Organotypic slice culture and immunocytochemistry

250 µm sagittal slices of cerebellar cortex were made from P7 mice using a vibratome (Leica VT 1000S) [67,68] and treated with WNT3 (100 ng/ml) or control supernatant for 24 h. For immunocytochemistry, organotypic slice cultures were fixed with 4% paraformaldehyde, incubated in 20% sucrose for 24 h, and embedded in OCT (Tissue Tek). Cryostat sections (16 µm) were generated with a Leica CM30505 cryostat, after which sections were permeabilized in 0.5% Triton X-100/PBS, blocked in 10% normal goat serum/PBS and immunostained with primary and secondary antibodies as described above. Confocal images were acquired with a Radiance 2000 confocal laser-scanning microscope (Bio-Rad). 

### Luciferase reporter assay

The luciferase vectors we used included a Firefly luciferase plasmid controlled by the TCF/LEF promoter (pTOP-flash) to study Wnt-β-catenin signaling pathway, a Firefly luciferase plasmid (SRE) driven by a minimal MAPK promoter plus multiple SRE binding sites to test MAPK signaling pathway, and a Renilla luciferase vector driven by the TK promoter (pRL-TK) to normalize the results of Firefly luciferase activity assays. The vectors were electroporated into 5x10^6^ purified GCPs, using the Amaxa mouse neuron nucleofection kit (Amaxa Biosystems). The transfection included 1 µg of SRE-Firefly-luciferase vector and 75 ng of Renilla-TK vector (Stratagene). 1x10^6^ cells were then plated in 96 well dishes, and when indicated, WNT3 was added at 100 ng/ml, SHH (R&D) at 3 μg/ml, BDNF (R&D) at 10 ng/ml and PD98059 (Cell Signaling) at 50 μM for 24 h post-transfection. To measure the luciferase activity, cells were lysed and the luciferase assay was performed with the Dual-Luciferase Reporter Assay (Promega) as described by the manufacturer. 

#### Culture of mouse medulloblastoma cells in vitro

To make sure that the loss of p53 did not affect the response of tumor cells to WNT3 treatment, tumor cells were purified by Percoll density gradients from medulloblastomas that spontaneously arose from two tumor-predisposed genetically engineered mouse models of SHH medulloblastoma, *Ink4c-/-, p53Fl/-, Nestin-Cre+* and *Ink4c-/-, Ptch1+/-* [23]. Tumor cells were grown on PDL and MATRIGEL coated plates in Neurobasal medium with B27, N2, 2mM glutamine, Pen/Strep (Invitrogen), 4 mg/ml BSA, and 0.45% D-Glucose. Cyclopamine was used at 10 μM (LC laboratories), in the presence or absence of 100 ng/ml of WNT3 supernatant.

#### Propagation of flank allografts

Medulloblastoma cells were purified by Percoll density gradient from five independent primary tumors that spontaneously arose from *Ink4c-/-, p53Fl/-, Nestin*-*Cre+* mice. Tumor cells purified from each tumor were pooled and infected with MSCV-based retroviruses either expressing only the yellow fluorescent protein (YFP) from an internal ribosomal entry site or co-expressing WNT3 and YFP. 0.5 x 10^6^ infected cells were either directly subcutaneously transplanted into the flank of six naïve recipient nude mice or FACS sorted for YFP positive cells before transplant into the flank of four recipient nude mice (2 mice per tumor). Secondary allograft flank tumors were analyzed 3 weeks later by weight and for YFP expression. To control for variability, flank injections of YFP only marked tumor cells was done on the right side while tumor cells expressing WNT3 and YFP were transplanted on the left flank of each recipient mouse. 

### Flow cytometry, cell cycle and apoptosis analyses

YFP expression and DNA content were measured by fluorescent activated cell sorting (FACS), while apoptosis was analyzed by staining with Annexin-V antibody (Roche).

### Statistical analysis

Data were presented as mean ± s.e.m. from at least three independent experiments. Direct comparisons were made using Student’s t-test and multiple group comparisons were made using one-way analysis of variance (ANOVA). Statistical significance was defined as P < 0.05, 0.01 or 0.001 (indicated as *, ** or ***, respectively). P values ≥ 0.05 were considered not significant.

## Results

### WNT3 decreases proliferation and promotes differentiation of GCPs

To investigate the role of the *Wnts* in cerebellar development, we assayed the expression of ten *Wnt* family members, *Wnt1, Wnt3, Wnt3a, Wnt4, Wnt5a, Wnt5b, Wnt6, Wnt7a, Wnt8b* and *Wnt10b*, in the mouse cerebellum from postnatal day 5 (P5) to adult by quantitative PCR (qPCR). These assays revealed expression of *Wnt3, Wnt4, Wnt5a, Wnt5b, Wnt7a*, and *Wnt10b* mRNA transcripts during cerebellar development, whereas *Wnt1, Wnt3a, Wnt6* and *Wnt8b* transcripts were not detected (Figure 1A). Of the WNTs expressed during cerebellar development, only *Wnt3* and *Wnt7a* levels increased postnatally into adulthood, at a time when GCPs exit the cell cycle, differentiate and mature. As WNT7a has been characterized extensively, with prior experiments demonstrating a role for WNT7a in synapse formation [37,38,39,40], we focused on the role of WNT3 in GCP development. Note that WNT3 protein levels also increased postnatally (Figure 1B). 

In order to test the effects of WNT3 on purified GCPs *in vitro* or on GCPs in organotypic slice preparations, we prepared enriched WNT3 from conditioned medium. WNT proteins are lipid-modified glycoproteins that need to be solubilized in medium containing hydrophobic carriers for optimal activity, and using conditioned medium is a classical approach for testing WNT function [38,69]. To produce WNT3, we stably infected NIH3T3 cells with the retroviral vector *pCXi* containing *Wnt3* cDNA that expresses the secreted WNT3 protein. A *pCXi* control vector was also transfected into NIH3T3 cells to obtain control conditioned medium. Enriched WNT3 was analyzed by immunoblotting for WNT3 expression (Figure S1A), and the concentration of WNT3 in the conditioned medium was estimated at 1 µg/ml (data not shown). Since the concentration of WNT3 in early postnatal cerebellum was estimated at 100 ng/ml by immunoblotting cerebellar lysates for WNT3 expression (unpublished data), we treated purified GCPs and cerebellar slices with 100 ng/ml of WNT3, and the same volume of control medium was used for comparison. 

**Figure 4:**
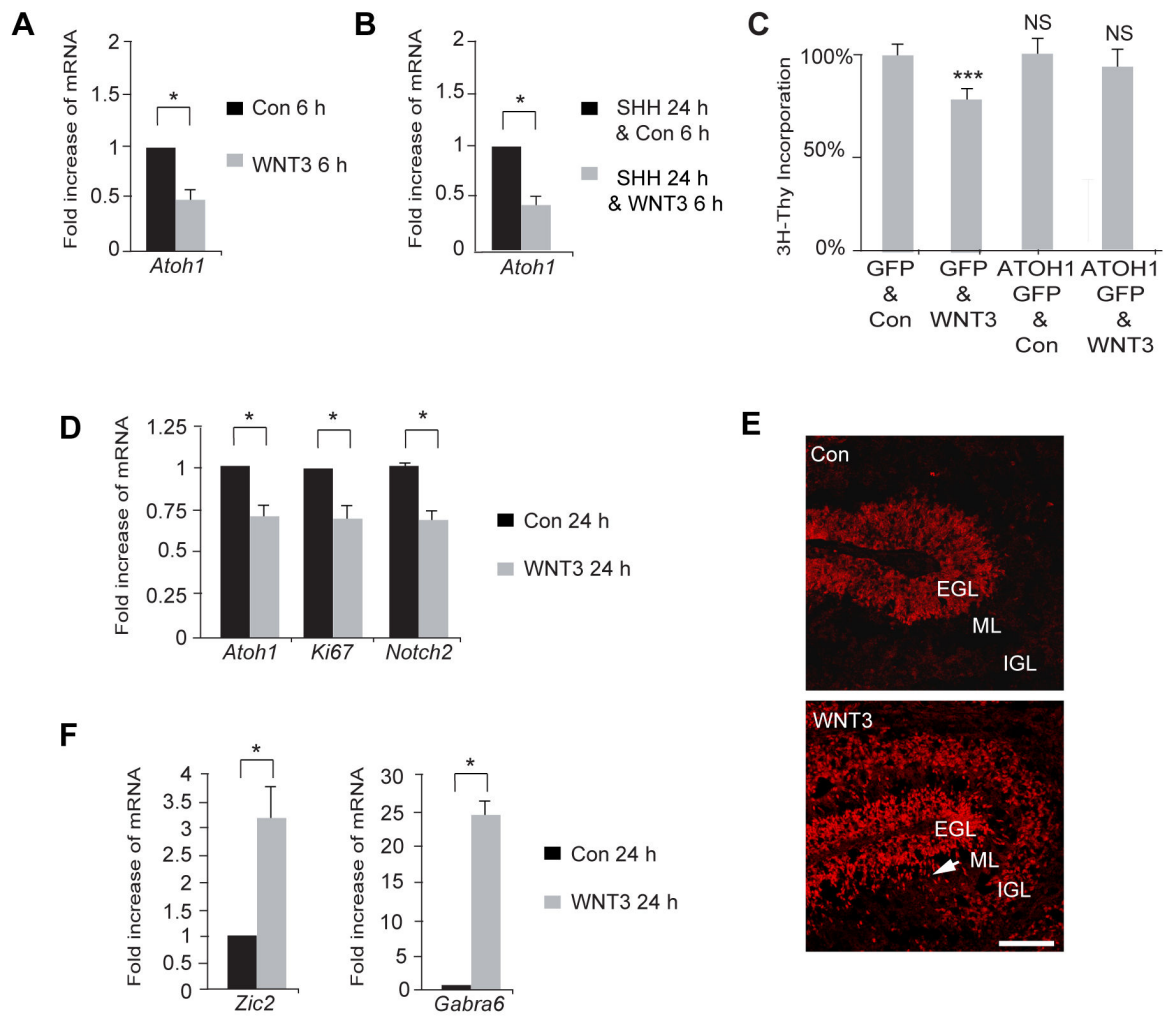
GCP proliferation induced by WNT3 is not dependent on the BMP pathway. (*A*) WNT3 and BMPs (BMP2, BMP4, BMP6, and BMP7) cooperated to decrease proliferation of GCPs as measured by [^3^H]-Thymidine incorporation assay. (*B*) The BMP inhibitor Noggin did not affect the WNT3-mediated decrease in proliferation. (WNT3, 59.9±4.6% of the control value; Con/Noggin, 93.5±1.7%; and WNT3/Noggin 46.4±2.9% (n=3).) (*C*) GCPs were stimulated for 4h at 1 DIV with WNT3 or BMP7 and analyzed by immunoblotting with the anti-phospho-SMAD1/5/8 antibody. The anti-SMAD1 antibody was used for normalization to obtain the percentage of phosphorylated SMAD1. (Con, 100±8.3%; WNT3, 133.8±33.8%; Con, 100±11.5%; and BMP7, 373.5±88.9% (n=3). Data represent the mean ± s.e.m.: *p<0.05, **p<0.01, ***p<0.001. NS, not significant.

The addition of WNT3 to cultures of GCPs purified from P6 cerebella for 24 h inhibited cell proliferation, measured by [^3^H]-Thymidine incorporation, by approximately 40% relative to control cells (Figure 2A), which was similar in degree to that of BMP treatment of GCPs (Figure 4A). We also tested newly commercially available recombinant WNT3 in our growth assay and found that this recombinant WNT3 also decreased GCP proliferation (Figure S4A). TUNEL assays did not reveal a significant difference in the percentage of apoptotic cells between control and WNT3-treated cultures, suggesting that changes in cell number we observed after treatment with WNT3 did not relate to cell death (Figure 2B).

As SHH drives the proliferation of GCPs during postnatal cerebellar development [1], we examined whether WNT3 can antagonize the mitogenic effect of SHH by treating purified GCPs with SHH (3 µg/ml) or with both WNT3 and SHH (Figure 2C). The results of these experiments showed that treatment with WNT3 significantly reduced SHH-induced GCP [^3^H]-Thymidine incorporation relative to levels observed in controls treated with SHH alone (Figure 2C). This suggests that WNT3 is a negative regulator of GCP proliferation that can antagonize the mitogenic action of SHH. Since SHH drives GCP proliferation by inducing pro-proliferative genes that include *Gli1*, *Gli2*, the *D-type cyclins*, and *Mycn* [23,70,71,72], we measured the levels of the SHH-dependent transcriptional targets *Gli1*, *Gli2*, *Mycn*, *Ptch1*, and *Ccnd1* by qPCR (Figure 2D,E). The levels of *Gli1*, *Gli2*, and *Ptch1* genes were decreased after applying WNT3 to SHH treated GCPs (Figure 2E). Similar changes were seen in the presence of cycloheximide, a protein synthesis inhibitor, suggesting that down-regulation of these genes by WNT3 did not depend on new protein synthesis (Figure S1B). These results suggest that WNT3 inhibits GCP proliferation, at least in part, by down-regulating the levels of a subset of SHH downstream target genes. 

To determine the level at which WNT3 impinges on the SHH pathway, we examined whether WNT3 acts upstream or downstream of the transcriptional regulator GLI1. We electroporated GCPs with a pSR *Gli1*-expressing vector, treated cells with control medium or WNT3, and examined the mRNA transcript levels of Hh target genes by qPCR. Overexpression of GLI1 in GCPs resulted in a 320 fold (± 119) and 460 fold (± 166) increase in *Gli1* transcript levels in the control and WNT3 conditions, respectively. In addition, overexpression of GLI1 increased the transcript levels of the HH target genes *Gli2 and Ptch1*. In contrast, the transcript levels of these target genes were not upregulated by GLI1 overexpression in the presence of WNT3 (Figure 2F), confirming that WNT3 inhibits HH signaling at the level or downstream of the transcriptional activator GLI1. 

**Figure 3:**
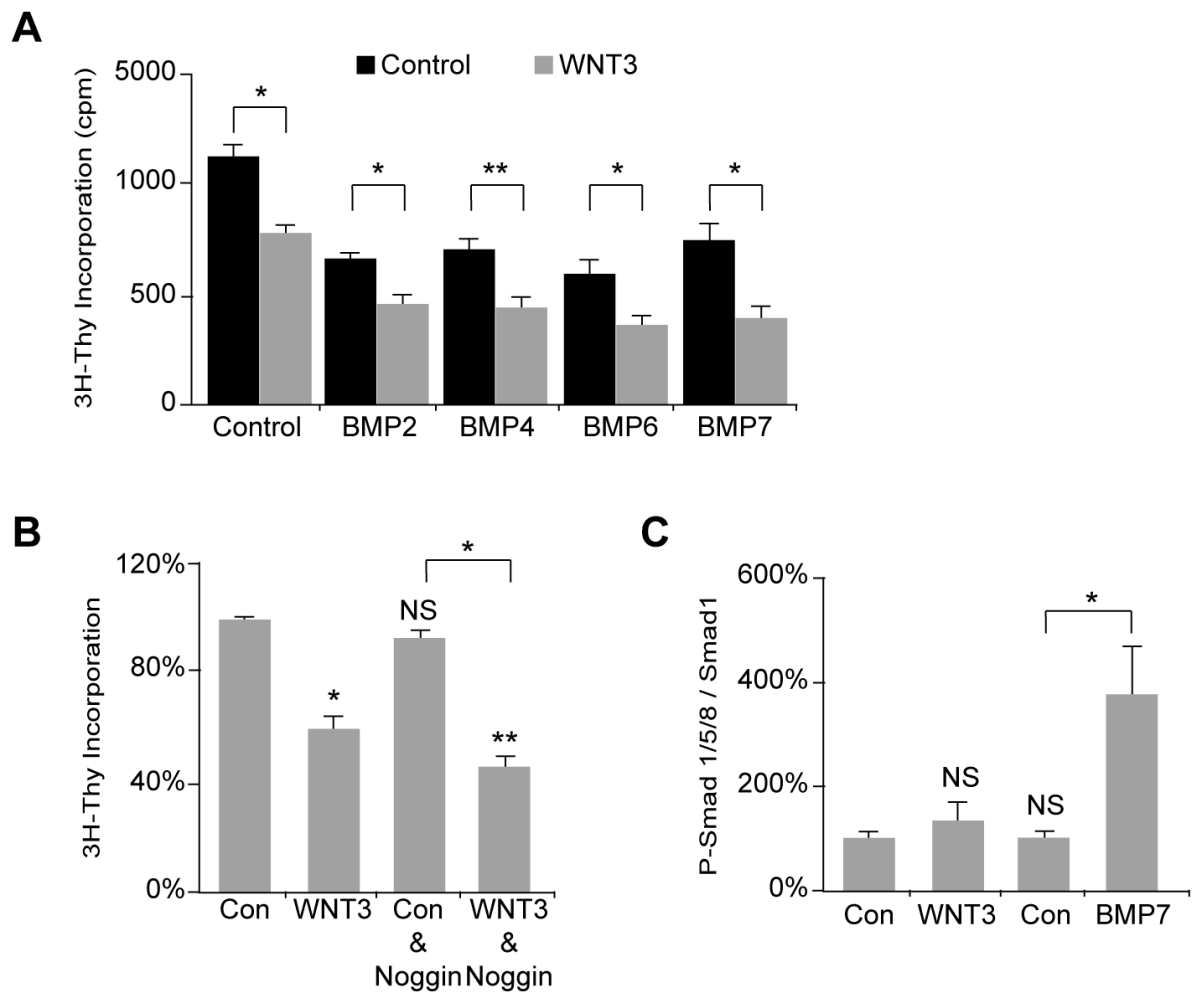
WNT3 decreases the GCP proliferation marker *Atoh1* and increases the GCP differentiation marker PAX6. (*A*) WNT3 decreased *Atoh1* mRNA levels after 6 h of treatment (Con=1; WNT3: *Atoh1*=0.52±0.06). (*B*) Treatment of GCPs with WNT3 decreased *Atoh1* mRNA levels in the presence of SHH. SHH was added for 24 h and WNT3 was added for 6h. (SHH/Con=1; SHH/WNT3: *Atoh1*=0.47±0.04). (*C*) Overexpression of ATOH1 by retroviral infection of GCPs abrogated the WNT3 effect as measured by [^3^H]-Thymidine incorporation assay. GCPs were infected with retroviruses produced using pMSCV-GFP and pMSCV-*Atoh1-GFP* on day 1. WNT3 was added to the GCPs on day 2. (GFP/Con, 100±2.5%; GFP/WNT3, 77.4±3.3%; ATOH1-GFP/Con, 98.9±2.3%; ATOH1-GFP/WNT3, 92.6±3.8% (n=3).) (*D*) WNT3 decreased the mRNA levels of additional mitotic markers Ki67 and *Notch2* after 24 h of treatment (Con=1; WNT3: *Atoh1*=0.71±0.07, Ki67=0.70±0.08, and *Notch2*=0.70±0.06). (*E*) P7 cerebellar slices were incubated with WNT3 for 24 h, and cryostat sections were immunostained with anti-PAX6 antibody, a marker for GCP differentiation. In control cultures (top panel), low levels of immunostaining are seen with anti-PAX6 antibody. WNT3 treatment (lower panel) increases the intensity of anti-PAX6 immunostaining in GCPs in the external granule layer (EGL), in postmitotic GCPs migrating across the molecular layer (ML, arrow) and in granule neurons in the internal granular layer (IGL) undergoing terminal differentiation. (*F*) WNT3 increased the mRNA levels of additional post-mitotic markers Zic2 and *Gabra6* after 24 h of treatment (Con=1; WNT3: Zic2=3.22±0.54 and *Gabra6*=24.40±2.08). Data represent the mean ± s.e.m.: *p<0.05, **p<0.01, ***p<0.001. NS, not significant. Scale bar 100 µm.

We next examined whether treatment with WNT3 affected the expression level of markers for proliferating GCPs. In the developing cerebellum, the bHLH transcription factor *Atoh1* is essential for GCP neurogenesis [73,74], and provides a marker for proliferating GCPs. By qPCR, we observed that WNT3 down regulates the levels of *Atoh1* (Figure 3A,B). To test whether reduced expression levels of *Atoh1* contribute to the decreases in GCP proliferation we observed, we over-expressed *Atoh1* by retroviral infection (Figure S1C) and assayed proliferation in the presence and absence of WNT3 with a [^3^H]-Thymidine incorporation assay. Over-expression of ATOH1 suppressed the growth inhibition observed with WNT3 relative to results with a control retrovirus (Figure 3C). Thus, the suppression of GCP proliferation by WNT3 involves down-regulation of *Atoh1*. The mRNA expression of other mitotic markers, *Ki67* and *Notch2*, were also decreased with WNT3 treatment (Figure 3D). 

To assess whether the decrease in GCP proliferation by WNT3 is accompanied by an increase in differentiation, we examined the expression of GCP differentiation markers in WNT3 treated GCPs. We first examined the transcription factor PAX6, which functions in cerebellar granule neuron development [75,76,77] and serves as a marker for differentiated GCPs. We used immunocytochemistry to examine the effect of WNT3 treatment on the expression of PAX6 in cerebellar slices (Figure 3E). These experiments showed that WNT3 treatment, compared to control treatment, increased the intensity of immunostaining with antibodies against PAX6 in both the lower EGL, where GCPs are exiting the cell cycle, and in the IGL, where post-migratory granule neurons form synaptic connections with afferent mossy fibers. In addition, intensely labeled PAX6 cells were located in the molecular layer, where post-mitotic, differentiating GCPs extend parallel fiber axons and migrate along Bergmann glial processes. We also looked at the post-mitotic markers *Zic2* and *Gabra6* by qPCR, and found that the levels of these differentiation markers also increased with WNT3 expression (Figure 3F). Taken together, these results suggest that WNT3 inhibits GCP proliferation and promotes differentiation.

 Because BMP signaling pathways play a critical role in GCP differentiation [66,78,79], we next examined whether the GCP growth suppression of WNT3 involved BMP signaling. In agreement with prior studies, BMP2, BMP4, BMP6 and BMP7 inhibited [^3^H]-Thymidine incorporation (Figure 4A), and the addition of WNT3 further inhibited GCP [^3^H]-Thymidine incorporation (Figure 4A). The addition of the BMP antagonist Noggin did not alter WNT3-induced inhibition of GCP proliferation (Figure 4B), suggesting that WNT3 inhibition of GCP proliferation is independent of BMP signaling. To further examine whether WNT3 treatment activates BMP signaling, we assayed SMAD phosphorylation in GCPs treated with WNT3 or BMP7. Although treating GCPs with BMP7 triggered robust SMAD phosphorylation, no significant changes in SMAD phosphorylation occurred in GCPs treated with WNT3 (Figure 4C). These results suggest that inhibition of GCP growth by WNT3 is independent of BMP signaling.

### WNT3 suppresses medulloblastoma formation in a mouse model

**Figure 5:**
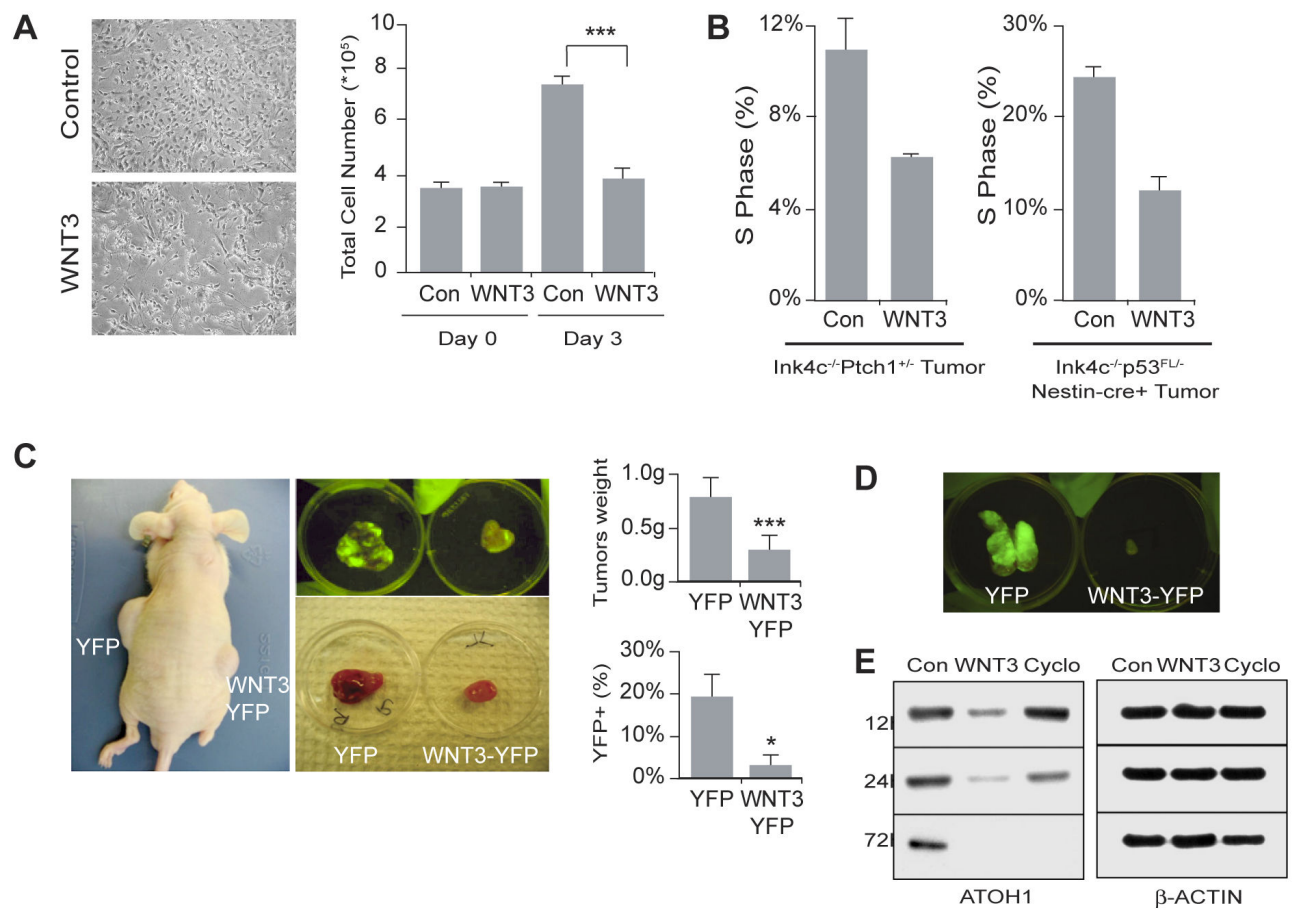
WNT3 inhibits murine medulloblastoma formation. (*A*) (Left panels) GCP-like tumor cells treated with WNT3 for 3 DIV. (Right panel) Quantitation of total cell number. (Day 0, Con and WNT3, 3.5x10^5^ cells; Day 3, Con, 7.7±0.3x10^5^ cells and WNT3, 3.8±0.3x10^5^ cells (n=5).) Scale bar 50 µm. (*B*) Cell cycle analysis by FACS of GCP-like tumor cells treated with WNT3 for 72 h. (Ink4c^-/-^Ptc1^+/-^ tumor, Con, 11±1.4% and WNT3, 6±0% (n=2) and *Ink4* ^*c-/-*^ *p53* ^*Fl/-*^ *Nestin-cre* ^+^ tumor, Con, 24.5±2.12%, and WNT3, 12.5±2.12% (n=2).) After treatment with WNT3, no increase in apoptosis was observed using Annexin V (data not shown). (*C*) (Right image panels) Image of allografts established from medulloblastoma cells of a mouse (Ink4c^-/-^p53^Fl/-^Nestin-cre^+^) infected with control virus expressing YFP only or with virus expressing WNT3 and YFP. (Top right graph) Weight of allografts derived from cells infected with control YFP (0.82±0.16 g) or with WNT3 and YFP (0.30±0.13 g) (n=4). (Bottom right graph) Analysis by FACS of YFP expression in allografts derived from cells infected with control YFP (19.7±4.1%) or with WNT3 and YFP (2.9±1.2%) (n=3). (*D*) Tumors from athymic mice bearing allograft from FACS-sorted YFP-positive tumor cells infected with control virus (top panel, YFP) or WNT3 (bottom panel, WNT3-YFP), (n=2). (*E*) ATOH1 protein was decreased in WNT3 treated GCP-like tumor cells. Cyclopamine was used as a postitive control. β-actin was used as a loading control. Data represent the mean ± s.e.m.: *p<0.05, **p<0.01, ***p<0.001. NS, not significant.

Because WNT3 decreased GCP proliferation, we tested whether WNT3 also affected the formation of medulloblastoma in two mouse models. GCP-like tumor cells were purified from tumors that spontaneously arose in *Ink4c* ^*-/-*^ *, p53* ^*Fl/-*^ *, Nestin-cre+* and *Ink4c* ^*-/-*^ *, Ptch1* ^*+/-*^ mice [23] and cultured for 3 days in the presence or absence of WNT3. Cells showed decreased growth in the presence of WNT3 (Control, 7.7±0.3x10^5^ cells and WNT3-treated, 3.8±0.3x10^5^ cells; Figure 5A). After treatment with WNT3, no increase in apoptosis was observed using Annexin V (data not shown). FACS analysis revealed a reduced number of cells in S phase (Figure 5B). When GCP-like tumor cells were incubated with WNT3, we noted increased expression of TAG1 and TUJ1, markers of differentiation (Figure S2). To assess whether WNT3 suppresses tumor growth *in vivo*, we infected tumor cells with retroviruses expressing YFP alone or WNT3 and YFP (WNT3-YFP) and injected them sub-cutaneously in the flank of athymic nude mice. Tumors derived from cells expressing WNT3-YFP were smaller than the tumors derived from cells expressing YFP alone (Figure 5C, bottom right image panel and top right graph). WNT3 YFP-expressing tumors also contained a reduced number of YFP positive cells, suggesting that the decrease in tumor growth was due to a decrease in proliferation of WNT3-YFP infected cells (Figure 5C, top right image panel and bottom right graph). Similar results were obtained when YFP positive cells selected by FACS were injected into athymic nude mice, indicating that WNT3 inhibited tumor growth (Figure 5D). To assess whether the inhibition of tumor growth involved changes in the levels of proteins important for GCP development, we assayed expression of ATOH1. As observed with cerebellar GCPs, treatment of medulloblastoma cells with WNT3 decreased the expression of ATOH1 (Figure 5E). Thus WNT3 is a potent inhibitor of medulloblastoma development.

### WNT3 activates the MAPK/ERK1,2 and ERK5 signaling pathway(s) in GCPs

**Figure 6:**
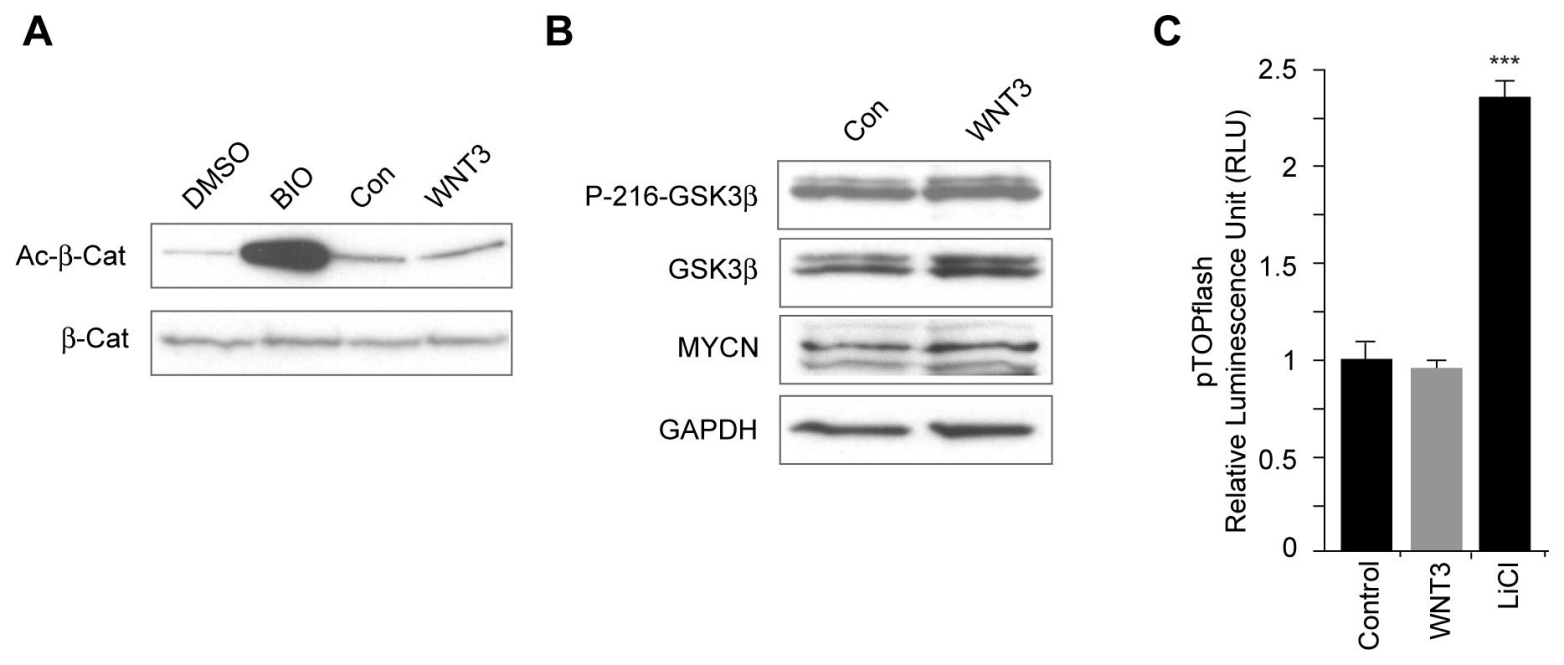
WNT3 signaling is not mediated by β-catenin signaling. (*A*, *B*,) GCPs were treated with WNT3 for 24 h and lysates analyzed by Western blot analysis. (*A*) Immunoblotting of GCP lysates with anti-active-β-catenin (Ac-β-cat) antibody showed that WNT3 did not induce β-catenin activation. The GSK-3 inhibitor BIO was used as positive control to increase activated β-catenin and activated β-catenin levels were compared to total β-catenin levels, using anti-β-catenin antibody (n=3). (*B*) Immunoblotting using anti-Phospho(P)-216-GSK-3β, anti-GSK-3β and anti-MYCN antibodies showed that WNT3 does not regulate β-catenin signaling. GAPDH was used as loading control. (*C*) Using a Luciferase assay, WNT3 fails to activate β-catenin signaling, which is reported by the expression of pTOPflash luciferase driven by the TCF/LEF promoter. LiCl was used as a positive control to activate β-catenin signaling. Data represent the mean ± s.e.m.: *p<0.05, **p<0.01, ***p<0.001. NS, not significant.

To define the signaling pathways involved in WNT3 inhibition of GCP and medulloblastoma growth, we examined whether WNT3 activates the canonical WNT/β-catenin signaling pathway. To investigate whether WNT3 affects β-catenin activity in GCPs, we compared β-catenin activity in GCPs treated with control or WNT3 to that in cells treated with the GSK-3 inhibitor 6-bromoindirubin-3'-oxime (BIO) [80]. The results of these experiments indicate that BIO, but not WNT3, increased the amount of activated β-catenin in lysates of GCPs (Figure 6A). In agreement with these findings, we found that WNT3 treatment did not affect the levels of active, tyrosine-216 phosphorylated GSK-3β, nor did it induce expression of the canonical WNT/β-catenin target MYCN in GCPs (Figure 6B). Since β-catenin interacts with TCF/LEF to control transcription, we also examined canonical WNT/β-catenin activity using a luciferase assay, in which we expressed pTOPflash luciferase driven by the TCF/LEF promoter. pTOPflash luciferase activity did not increase after treatment of GCPs with WNT3 compared to the control (Figure 6C and Figure S3A), suggesting that WNT3 does not activate canonical WNT/β-catenin signaling in GCPs. 

**Figure 7:**
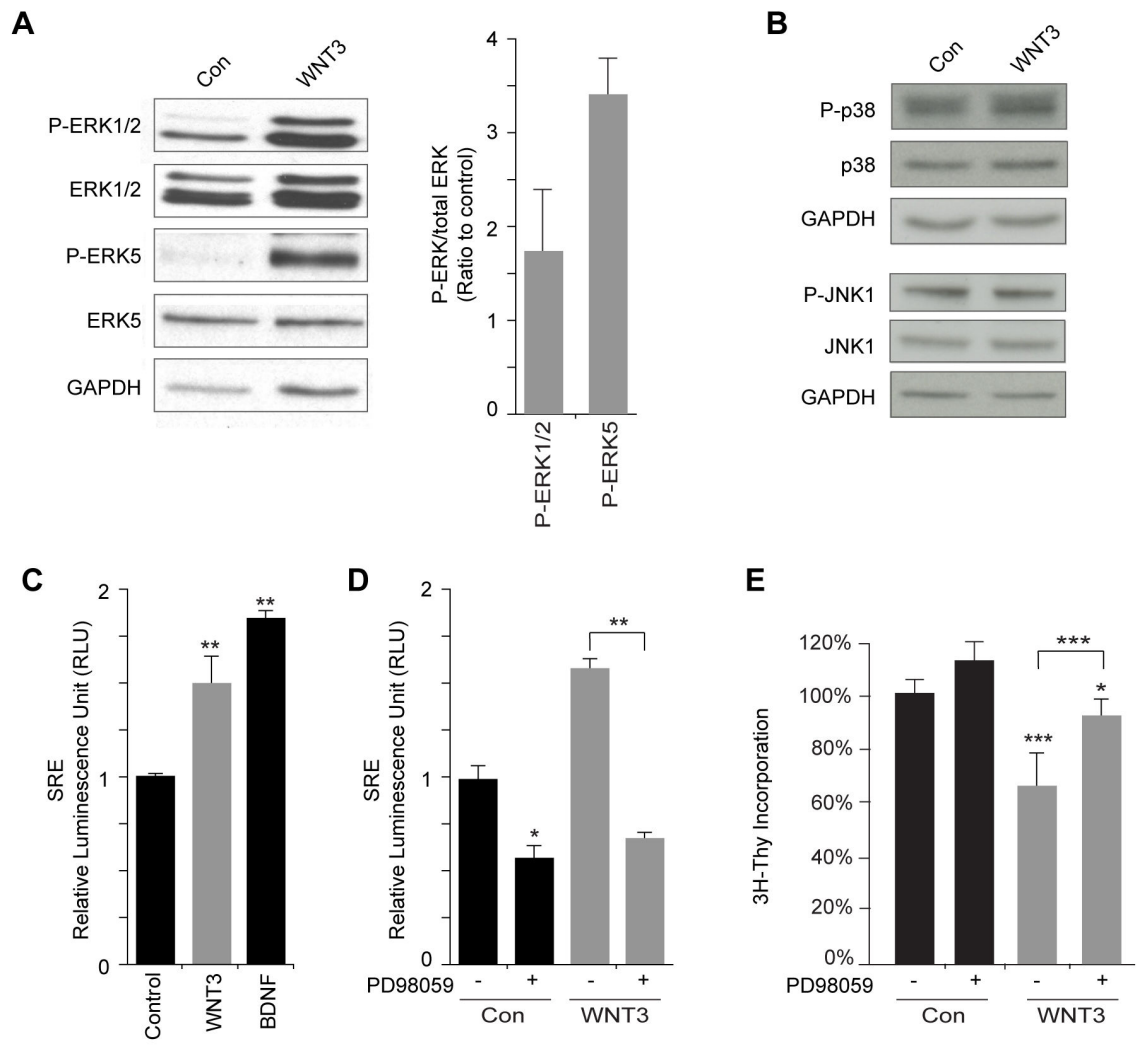
WNT3 activates the MAPK/ERK1,2 and ERK5 signaling pathway(s) in GCPs. (*A*,*B*) GCPs were treated with WNT3 for 24 h and lysates analyzed by Western blot analysis. (*A*) WNT3 increases ERK1/2 and ERK5 phosphorylation in GCPs. Protein extracts were analyzed by immunoblotting with anti-P-ERK1/2, anti-ERK1/2, anti-P-ERK5, and anti-ERK5 antibodies (n=3). GAPDH was used as loading control. (*B*) Immunoblotting using anti-P-p38, anti-p38, anti-P-JNK and anti-JNK antibodies showed that WNT3 did not alter p38 or JNK activity. GAPDH was used as loading control. (*C*) Test of WNT3 specificity using a SRE Luciferase assay in GCPs. BDNF was used as a positive control regulator of MAPK signaling (n=3). (*D*) MAPK activity induced by WNT3 in GCPs is inhibited by the MEK inhibitor PD98059. SRE-luciferase assay in GCPs treated with control or WNT3, in the absence or presence of PD98059 (n=3). (*E*). The MEK inhibitor PD98059 reverses WNT3 inhibition of GCP proliferation. As measured by [^3^H]-Thymidine incorporation, PD98059 increases GCP proliferation by 15%±7%, WNT3 inhibits GCPs proliferation by 34±12% and PD98059 + WNT3 inhibits GCP proliferation by 7%±5% of the control (Con) (n=3). Data represent the mean ± s.e.m.: *p<0.05, **p<0.01, ***p<0.001. NS, not significant.

Because WNT3 did not activate canonical, β-catenin-dependent signaling in GCPs, we investigated the effect of WNT3 on non-canonical signaling pathways. Mammalian MAPKs are serine/threonine-specific protein kinases that respond to extracellular stimuli and regulate various cellular activities important for brain development [81,82]. MAPKs include ERK1/2, ERK5, p38 and JNK. To determine whether WNT3 activates any of these MAPKs, we treated GCPs with WNT3 for 0-24 h and assessed the phosphorylation of the MAPKs ERK1/2, ERK5, p38 and JNK by immunoblotting analysis. WNT3 treatment resulted in a rapid increase of P-ERK1/2 that was sustained up to 24 h relative to levels in untreated, control cells (Figure 7A and Figure S3B). We infer from these data that basal levels of ERK1/2 activation occur in proliferating GCPs and that changes in the intensity and duration of ERK1/2 activation induced by WNT3 shifted the cells toward differentiation. Similarly, very low levels of P-ERK5 detected in the control GCP population were also vastly increased by WNT3 treatment of GCPs (Figure 7A and Figure S3B). In contrast, WNT3 treatment did not increase the levels of P-p38 and P-JNK, compared to the control condition (Figure 7B). These results suggest that WNT3 activates ERK1/2 and ERK5, but does not increase the levels of P-p38 or P-JNK in GCPs, relative to controls. 

To corroborate that WNT3 treatment activated MAPK/ERK signaling, we used a plasmid with Firefly luciferase expression controlled by a minimal promoter containing multiple serum response factor (SRE) binding sites (Figure 7C). After incubating GCPs with WNT3 for 24 h, luciferase expression increased 1.5 ± 0.14 fold compared to control. BDNF was included as a positive control, as it has been shown to increase MAPK/ERK signaling. Notably, the newly commercially available recombinant WNT3 protein described above increased SRE luciferase activity, but not pTOPflash luciferase activity, similar to our WNT3 conditioned medium (Figure S4B,C).

To determine whether MAPK mediates the inhibitory effect of WNT3 on GCP proliferation, we inhibited MAPK activity using the MEK inhibitor PD98059. We first tested the efficacy of the inhibitor using the Firefly luciferase controlled by a minimal promoter with multiple SRE binding sites. After incubating GCPs with PD98059 for 24 h, endogenous MAPK activity was decreased compared to the control condition, and the increase in MAPK activity induced by WNT3 was completely inhibited (Figure 7D). Next, we treated GCPs with PD98059 for 24 h and measured proliferation by [^3^H]-thymidine incorporation. While treatment of GCPs with PD98059 only increased GCP proliferation slightly in the control condition, the MEK inhibitor totally reversed the inhibitory effect of WNT3 on GCP proliferation (Figure 7E). These results confirm that WNT3 inhibits GCP proliferation via activation of MAPK signaling. 

### WNT3 activates the MAPK/ERK signaling pathway(s) ex vivo in cerebellar organotypic slices and in medulloblastoma cells

**Figure 8:**
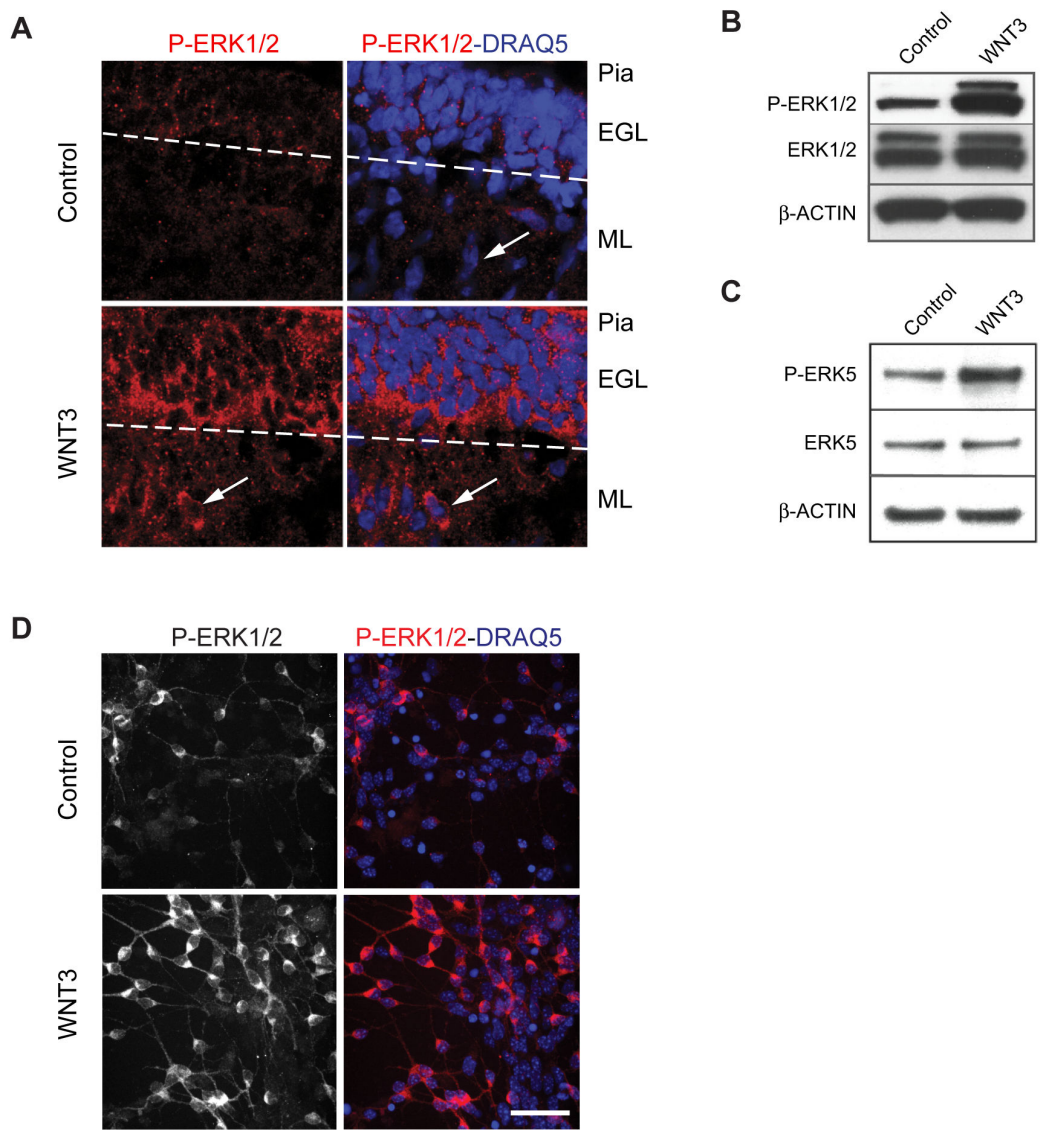
WNT3 induces activation of ERK1/2 *ex* *vivo* in P7 cerebellar slices and in medulloblastoma cells. (*A*) P7 cerebellar slices were incubated with WNT3 for 24 h, and cryostat sections were immunostained with anti-P-ERK1/2 antibodies. Nuclei were stained with DRAQ5. P-ERK1/2 was induced in the external granule layer (EGL), and localized in the cytoplasm of the GCPs in the lower aspect of the EGL, where GCPs commence differentiation. P-ERK1/2 was also located in the molecular layer (ML) where postmitotic GCPs migrate toward the internal granule layer (IGL). Arrows point to an individual cell in the ML to highlight the cytoplasmic localization of P-ERK1/2. Scale bar 20 µm. (B C, D) GCP-like tumor cells were treated 24 h with WNT3. After treatment, GCP-like tumors were lysed and analysed by immunoblotting using (*B*) anti-P-ERK1/2 and anti-ERK1/2 antibodies or (*C*) anti-P-ERK5 and anti-ERK5 antibodies. β-ACTIN was used as loading control. (*D*) GCP-like tumor cells were immunostained after treatment using anti-P-ERK1/2 antibodies. Nuclei were stained with DRAQ5. WNT3 treatment increased the number of GCP-like tumor cells containing cytoplasmic P-ERK1/2, compared to the control. Scale bar 20 µm.

P-ERK1/2 expression in P7 cerebellar cortex is highest in the inner aspect of the EGL, where GCPs exit the cell cycle and undergo differentiation (data not shown). To examine whether WNT3 treatment increased the phosphorylation of ERK1/2 *ex vivo*, we treated organotypic slices of P7 cerebellum with control or WNT3 for 24 h and assayed the levels of P-ERK1/2 immunostaining. WNT3 treatment increased the intensity of immunostaining with anti-P-ERK1/2 antibodies in the cytoplasm of GCPs located in the lower aspect of the EGL, a zone where GCPs differentiate, and in the ML where postmitotic GCPs migrate along Bergmann glial fibers (Figure 8A). These results indicate that WNT3 treatment activated MAPK/ERK signaling in GCPs located in zones where cells are exiting the cell cycle and differentiating. 

To examine whether WNT3 treatment activated ERKs in a mouse model of medulloblastoma, we measured P-ERK levels in purified tumor cells cultured for 24 h with WNT3 by immunoblotting and immunolabeling. Immunoblotting showed a marked increase in P-ERK1/2 and P-ERK5 levels with WNT3 treatment in medulloblastoma cells (Figure 8B,C). Immunolabeling with anti-P-ERK1/2 antibodies further showed that WNT3 treatment increased P-ERK1/2 in medulloblastoma cells (Figure 8D). These results highlight a common mechanism for inhibiting cell proliferation in normal CNS progenitors and in CNS neural tumors. 

## Discussion

**Figure 9:**
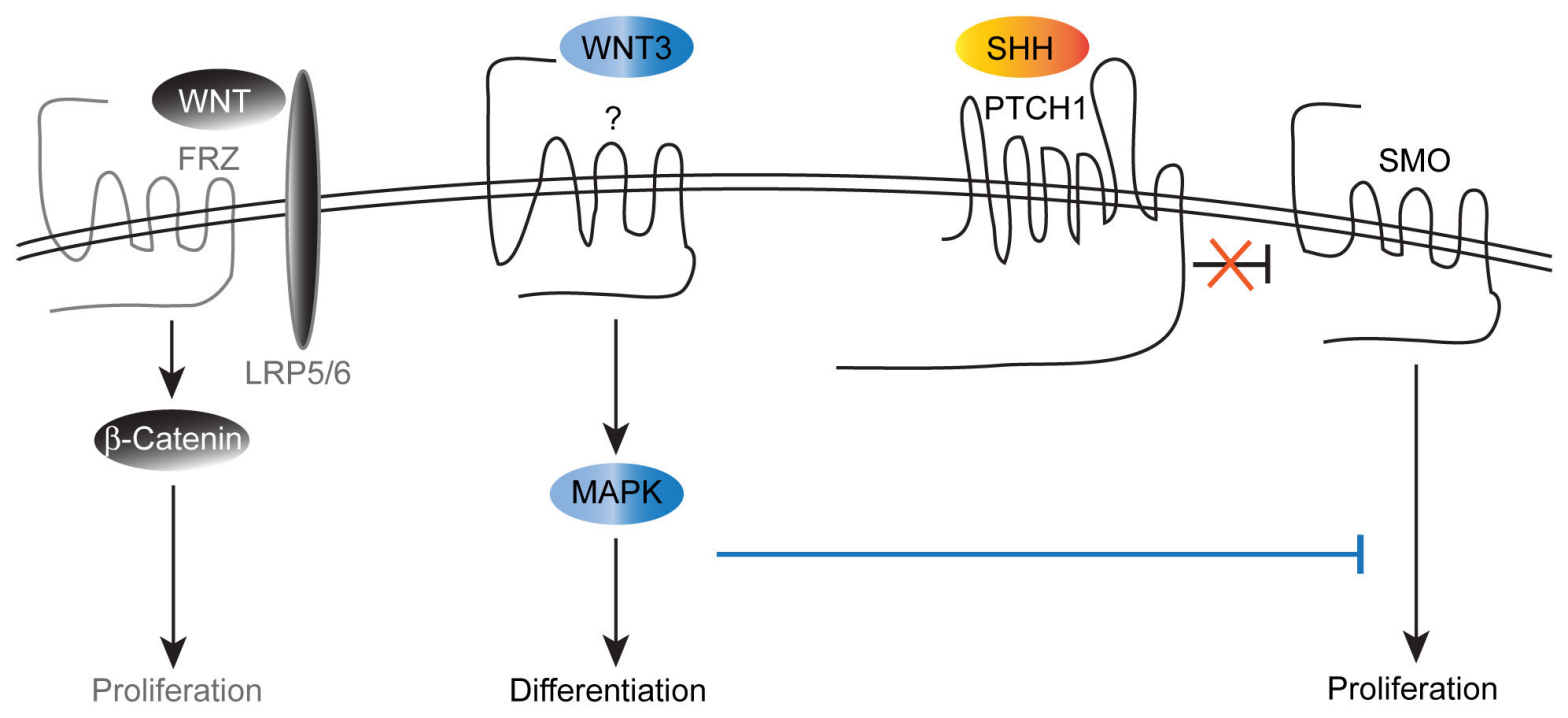
Interactions between WNT3 and SHH signaling pathways in GCPs. In Hedgehog (Hh) signaling, Sonic Hedgehog (SHH) binds Patched (PTCH) receptor, relieving its inhibition of Smoothened (SMO), and stimulating GCP proliferation. In WNT3 signaling, WNT3 binds an undefined receptor to activate MAPK signaling via a non canonical WNT pathway to promote GCP differentiation, whereas other WNTs bind Frizzled (FRZ) receptor to activate the canonical β-catenin pathway and stimulate proliferation. We propose a model whereby regulation of MAPK activity by WNT3 signaling alters the balance between proliferation and differentiation in GCPs, decreasing proliferation and increasing differentiation.

The present study identifies WNT3 as a novel extracellular regulator of GCP proliferation and differentiation during normal mouse cerebellar development, and as an inhibitor of tumor growth in a mouse model of medulloblastoma. WNT3-treatment inhibited GCP proliferation and antagonized the mitogenic growth stimulation of SHH by down-regulating SHH pathway components and the bHLH transcription factor *Atoh1*, which is highly expressed in proliferating GCPs. GCP growth suppression of WNT3 occurs through a non-canonical WNT pathway that activates MAPK ERK1/2 and ERK5 signaling (Figure 9). Importantly, WNT3 also suppressed medulloblastoma growth *in vivo* in a mouse model of medulloblastoma through mechanisms that involve activation of ERK1/2 and ERK5 signaling. 

In many organisms, *Wnt3* is expressed in the dorsal neural tube. In mouse, *Wnt3* is expressed in the dorsal part of the spinal cord, parts of the diencephalon, the dorsal thalamus, the midbrain-hindbrain region, dorsal ectoderm, branchial arches, limb buds, and differentiating motor neurons [83,84,85,86,87]. Although highly similar in sequence, WNT3 and WNT3a have been reported to play different roles during development [88,89], and a loss of function mutation of *Wnt3* cannot be rescued by *Wnt3a* [90]. Our analysis of *Wnt3* gene expression indicated that *Wnt3* mRNA, but not *Wnt3a*, is expressed in the developing cerebellum (Figure 1A), and WNT3 protein levels increase during the first two post-natal weeks (Figure 1B). Our results are in agreement with a prior study that reported expression of *Wnt3* by Purkinje neurons [91], which appear at E16.5 and mature postnatally when cerebellar granule cells exit the cell cycle and differentiate. Thus the timing of Purkinje neuron maturation, WNT3 expression, and GCP differentiation support the view that WNT3 inhibits GCP proliferation and promotes differentiation. Interestingly, SHH is also produced by Purkinje cells [1], but levels of SHH signaling components tend to decrease as cerebellar granule cells exit the cell cycle and differentiate (Figure S5). The differential expression profiles of WNT3 and SHH signaling components suggest that these signaling pathways contribute to the balance between GCP proliferation and differentiation during GCP development.

The present study indicates that WNT3 antagonizes SHH signaling by down-regulating pro-proliferative SHH target genes and shifts the GCP population toward differentiation, consistent with previous studies showing that WNT3 attenuates SHH signaling during chick neural tube patterning [85]. However, the ability of WNT3 to down-regulate a subset of SHH target genes, namely *Gli1*, *Gli2*, and *Ptch1*, but not *Ccnd1* or *Mycn* (Figure 2D,E), suggests that the effect of WNT3 on GCP growth is not merely due to an effect on proliferation, and could reflect context dependent, combinatorial regulation of Gli1-3 transcription factors and the contribution of other signaling pathways [92]. The growth inhibition of GCPs by WNT3 and decrease in SHH target genes is accompanied by a decrease in the transcription factor *Atoh1* (Figure 3A,B), a marker for proliferating GCPs, and an increase in the expression of the GCP differentiation marker PAX6 (Figure 3D), suggesting that WNT3 decreases GCP proliferation while promoting differentiation. While the extent of GCP growth inhibition that we observed with WNT3 (Figure 2A) is comparable to the level of inhibition reported for BMPs (Figure 4A) [66,79], WNT3 inhibits GCP growth independent of BMP signaling. This conclusion is based on our findings that the BMP antagonist Noggin did not alter WNT3-induced inhibition of GCP proliferation (Figure 4B) and WNT3 treatment did not activate SMAD signaling in the GCP population (Figure 4C). Notably, the degree of GCP growth inhibition that we observed with WNT3 (Figure 2A) is larger than the inhibition reported for pituitary adenylate cyclase-activating polypeptide (PACAP) [93], which also opposes the action of SHH signaling pathways on GCPs [93]. Importantly, both WNT3 enriched from conditioned medium and commercially available WNT3 decreased GCP proliferation and activated MAPK signaling (Figure 2A, Figure 7C, and Figure S4 A,C). Taken together, the results of the present study suggest that, in addition to integrating signals from SHH and BMPs, proliferating GCPs incorporate a WNT3 response to exit the cell cycle and begin to differentiate into granule neurons. Thus, multiple pathways control the remarkable expansion and differentiation of cerebellar GCPs in the early postnatal period. 

The non-canonical involvement of ERK signaling that we observed for WNT3 underscores the complexity of GCP development that involves multiple cellular processes and signaling pathways. We were initially surprised that WNT3 inhibition of GCP proliferation and mouse medulloblastoma formation involved activation of ERK1/2 and ERK5. However, a number of recent studies have reported a requirement for sustained ERK signaling in neuronal differentiation [94,95,96]. The MAPK/ERK signaling network coordinates inputs from different growth factors to regulate growth versus differentiation. Increasing evidence indicates that the role of ERK signaling in differentiation versus proliferation depends on the basal level of ERK activation, the nature of the stimulus and the duration of ERK activation. In PC12 cells, EGF and NGF shape MAPK network topology differently, eliciting positive or negative feedback loops and, in some cases, reversing specific responses to EGF or NGF [81]. Thus, the MAPK network in GCPs is likely to depend on growth factor context, with WNT3 as a novel MAPK regulator triggering sustained ERK signaling to induce cell cycle exit and/or differentiation. In GCPs, SHH signaling promotes proliferation [1], while WNT3 inhibits SHH transcriptional responses at the level of or downstream of the transcriptional activator GLI1 (Figure 2F), suggesting that a balance between these pathways determines whether cells will proliferate or differentiate (Figure 9). Interestingly, ERK5 has been implicated in the regulation of the pro-neural activities of Neurogenin1 (Ngn1) in cortical progenitors [97], which is consistent with our findings that P-ERK5 is present in GCPs and is associated with the differentiation of these neurons. These findings highlight the complicated nature of ERK signaling, and their potential involvement in multiple stages of GCP development and signaling pathways. Since the biological actions of ERKs depend on the cell type, the strength of stimulation, the duration of the signal and the subcellular localization of P-ERK [81,98], the precise role(s) that the ERKs play in GCP development will require further experimentation, and will be difficult to tease apart. 

At present, at least four families of proteins are thought to function as WNT receptors. Members of the Frizzled (FZD) protein family are well known for their ability to transduce canonical WNT/β-catenin signaling. The LDL receptor-related proteins, LRP-5 or LRP-6, act as co-receptors with FZD in canonical WNT/β-catenin signaling, arguing against a role for them in the effects of WNT3 on GCP proliferation [99,100,101]. The Receptor Tyrosine Kinase ROR2 acts as a receptor or co-receptor for WNT2, WNT5 and WNT5a [102,103]. Since ROR2 is not expressed in developing cerebellum [104], it can be excluded as a WNT3 receptor in cerebellar GCPs. The fourth class of WNT receptors is the Related to Receptor Tyrosine Kinase protein (RYK) [105,106,107]. Since RYK is expressed at high levels in the EGL of the developing mouse cerebellum (GENSAT project), where GCPs proliferate, exit the cell cycle, and commence differentiation, it is a putative receptor for WNT3 in cerebellar GCPs. Biochemical analyses of RYK as a WNT3 receptor are complicated by the finding that WNT/RYK signaling may require one of several co-receptors to promote differentiation (e.g. Ephrin receptors (Ephs)) [105,108]. Noteworthy, RYK interacts with Src family kinases (SFKs) [109], which contribute to Raf/ERK signaling. It is therefore plausible that WNT3 activates the RYK receptor to signal through a non-canonical Src/Raf/ERK pathway to modulate SHH signaling and thus the balance between proliferation and differentiation in developing GCPs [110,111]. Identification of the receptor(s) and co-receptor(s) that WNT3 acts through to regulate GCP proliferation and differentiation will provide further information on the mechanism(s) regulating these cellular processes.

These studies underscore the emerging view that the developing cerebellum provides a crucial system for evaluating molecular pathways that are important for the development of medulloblastoma [8]. The external germinal layer (EGL) of the early postnatal cerebellum is a complex structure that provides a system for analyzing the balance between the multiple pathways that govern the remarkable pace of GCP proliferation, increasing approximately 30 fold between birth and P15 (our unpublished observation), and induce cell cycle exit and differentiation. Although the complexity of studying cells in different stages of development poses technical challenges, it is of critical importance to discovering mechanisms of medulloblastoma formation, since subgroups of medulloblastoma arise from defects in signaling pathways that disrupt the normal balance of GCP growth and differentiation. Our finding that WNT3 is a potent suppressor of GCP growth and tumor formation in a mouse model of medulloblastoma suggests that WNT3 may have potential therapeutic value for the treatment of medulloblastoma in humans. Understanding how WNT3 responses are integrated with mitogenic signaling pathways, such as SHH and JAG1, and pathways that promote differentiation in GCP development and medulloblastoma, can be expected to provide critical insight on both normal cerebellar development and the growth of human medulloblastomas.

## Supporting Information

Figure S1 **(*A*) By Western blot analysis, a band with the apparent molecular weight of WNT3 is detected in conditioned medium collected from NIH 3T3 cells stably transfected with the pCXi retrovirus containing Wnt3 cDNA, but not from control NIH 3T3 cells.** (*B*) WNT3 decreased Gli1, *Atoh1*, and *Patched1* (Ptch1) mRNA levels following 6 h of WNT3 treatment in the presence of cycloheximide (CHX). Control=1, WNT3: Gli1=0.72±0.10, Gli2=0.93±0.05, *Atoh1*=0.62±0.08, Mycn=0.88±0.04, *Ptch1*=0.66±0.09, and *Ccnd1* =0.95±0.08. (*C*) Infection of GCPs with ATOH1 expressing retrovirus for 48 h increases the levels of ATOH1 2.06 fold over endogenous ATOH1 levels. Data represent the mean ± s.e.m.: *p<0.05, **p<0.01, ***p<0.001. NS, not significant.
(TIF)

Figure S2 **WNT3 increased TAG1 and TUJ1 levels in GCP-like tumor cells at 3 DIV.** Scale bar 100 µm.
(TIF)

Figure S3 **WNT3 regulation of MAPK signaling.** (*A*) Using a Luciferase assay, MAPK signaling increases proportionally to WNT3 concentration in GCPs, which is reported by the expression of Firefly luciferase driven by the SRE promoter, but WNT3 fails to activate β-catenin signaling, which is reported by the expression of pTOPflash luciferase driven by the TCF/LEF promoter. (*B*) Activation of ERK1/2 and ERK5 in response to WNT3 treatment in GCPs. ERK phosphorylation is rapidly induced with WNT3 treatment. GCPs were treated with WNT3 for 0 to 6 h, and lysates were analyzed by Western blot analysis, using anti-P-ERK1/2, ERK1/2, P-ERK5 and ERK5 antibodies. GAPDH was used as a loading control. (Bottom panels) Quantification of P-ERK1/2 and P-ERK5 intensity normalized to ERK1/2 and ERK5 signal, respectively, as a function of time.
(TIF)

Figure S4 **Commercially available, recombinant WNT3 inhibits proliferation in GCPs and activates MAPK signaling.** (*A*) Commercially available WNT3 decreases GCP proliferation by 18.6 ± 3.51% compared to the control (n=4) as measured by [^3^H]-Thymidine incorporation assay. Statistical significance of WNT3 or SHH is compared to control. (*B*) Using a luciferase assay, commercially available WNT3 fails to activate β-catenin signaling, which is reported by the expression of pTOPflash luciferase driven by the TCF/LEF promoter. CHIR99021 (136.53% ± 5.23% of control, n=3) is an inhibitor of glycogen synthase kinase 3β (GSK3β) that activates canonical WNT/β-catenin signaling, and was used as a positive control to demonstrate that the canonical WNT signaling pathway is functional in cultured GCPs. GCPs were treated with 250 ng/ml of commercially available WNT3 (Creative Biomart) or 3 µM CHIR99021 (StemGent). (*C*) Test of commercially available WNT3 specificity using an SRE luciferase assay in GCPs. Commercially available WNT3 (167.04 ± 10.04% of control, n=3) activates MAPK signaling, reported by the expression of Firefly luciferase driven by a minimal MAPK promoter plus multiple SRE binding sites. Data represent the mean ± s.e.m.: *p<0.05, **p<0.01, ***p<0.001. NS, not significant.
(TIF)

Figure S5 **qPCR of SHH signaling components, including *Gli1*, *Gli2*, *Ptch1*, *Ptch2*, *Smo*, *Ccnd1*, *Mycn*, and a transcription factor expressed in proliferating GCPs, *Atoh1*, in the mouse cerebellum from P5 to adult.** SHH signaling molecules and *Atoh1* levels were normalized against β-2-microglobulin (B2M), hyporanthine-guanine phosphoribosyltransferase (HPRT1) and ribosomal subunit 18s (M18s).
(TIF)

Table S1 **List of qPCR primers.**
(DOCX)
