# Supplementary figures and images for: WNT3 Inhibits Cerebellar Granule Neuron Progenitor Proliferation and Medulloblastoma Formation via MAPK Activation
Source: PLoS One. 2013 Nov 26;8(11):e81769. doi: 10.1371/journal.pone.0081769 (PMC3841149; doi:10.1371/journal.pone.0081769)

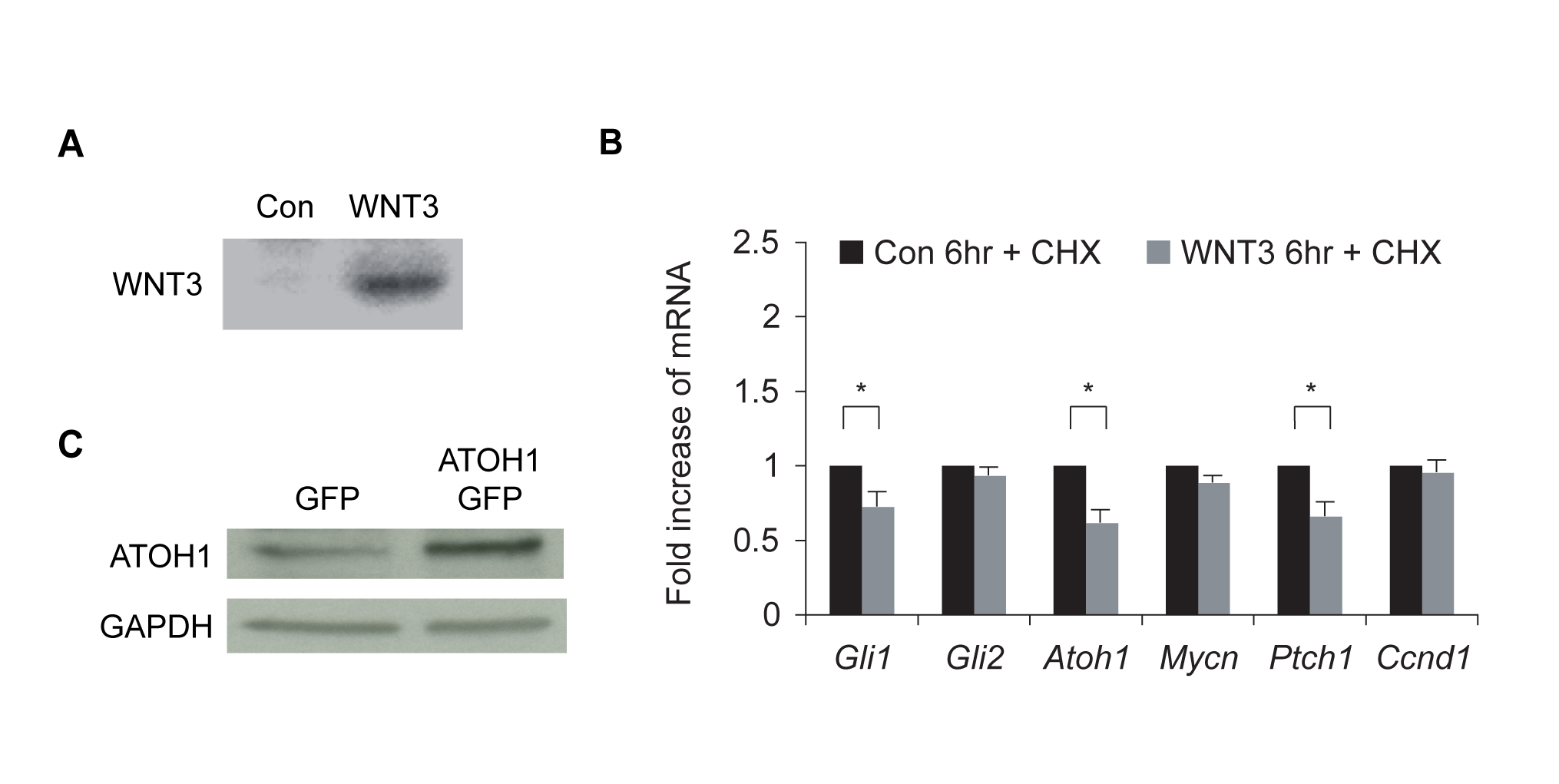

Supplement: Figure S1 — (A) By Western blot analysis, a band with the apparent molecular weight of WNT3 is detected in conditioned medium collected from NIH 3T3 cells stably transfected with the pCXi retrovirus containing Wnt3 cDNA, but not from control NIH 3T3 cells. (B) WNT3 decreased Gli1, Atoh1, and Patched1 (Ptch1) mRNA levels following 6 h of WNT3 treatment in the presence of cycloheximide (CHX). Control=1, WNT3: Gli1=0.72±0.10, Gli2=0.93±0.05, Atoh1=0.62±0.08, Mycn=0.88±0.04, Ptch1=0.66±0.09, and Ccnd1 =0.95±0.08. (C) Infection of GCPs with ATOH1 expressing retrovirus for 48 h increases the levels of ATOH1 2.06 fold over endogenous ATOH1 levels. Data represent the mean ± s.e.m.: *p<0.05, **p<0.01, ***p<0.001. NS, not significant. (TIF) [file pone.0081769.s001.tif]

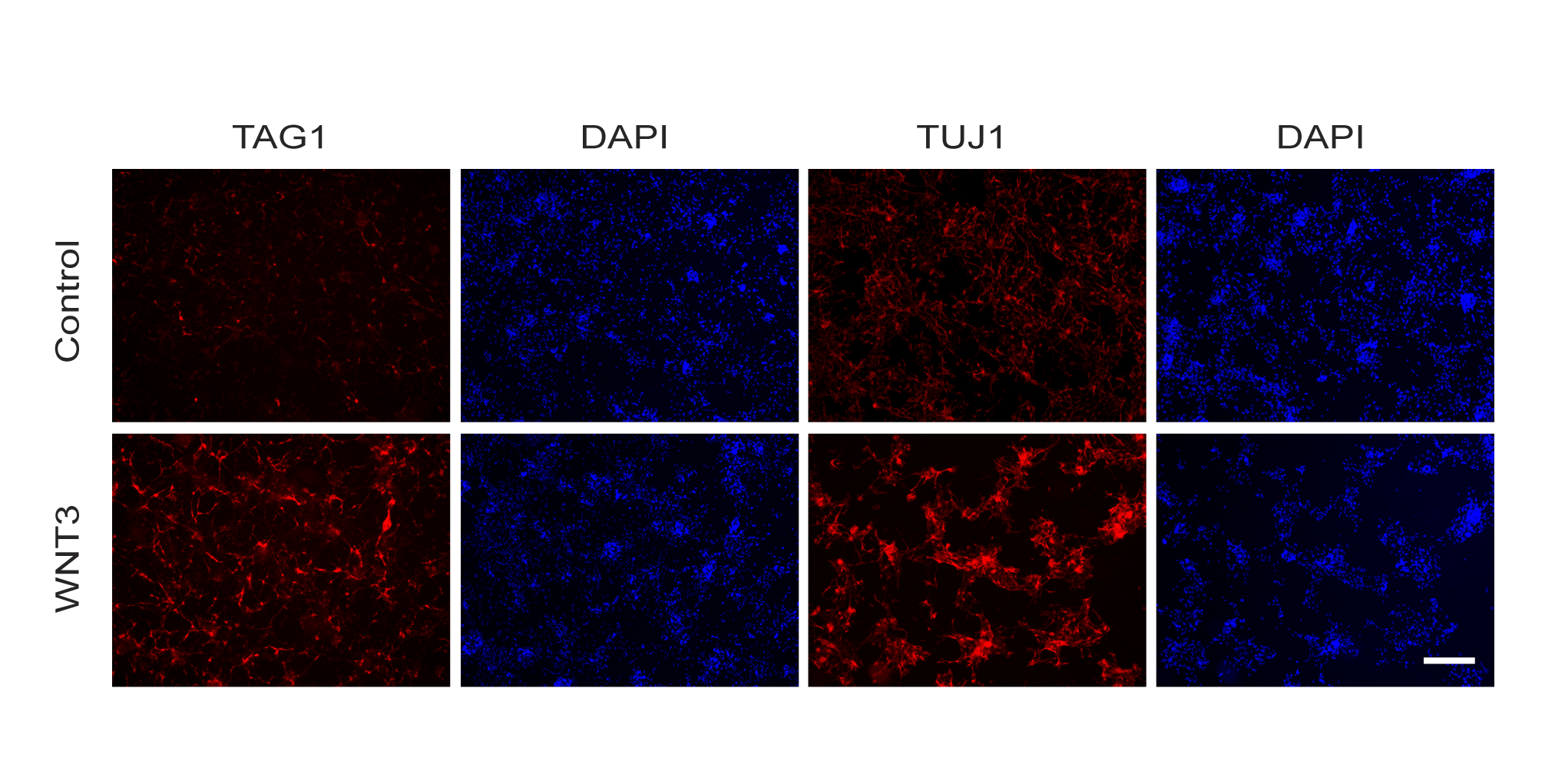

Supplement: Figure S2 — WNT3 increased TAG1 and TUJ1 levels in GCP-like tumor cells at 3 DIV. Scale bar 100 µm. (TIF) [file pone.0081769.s002.tif]

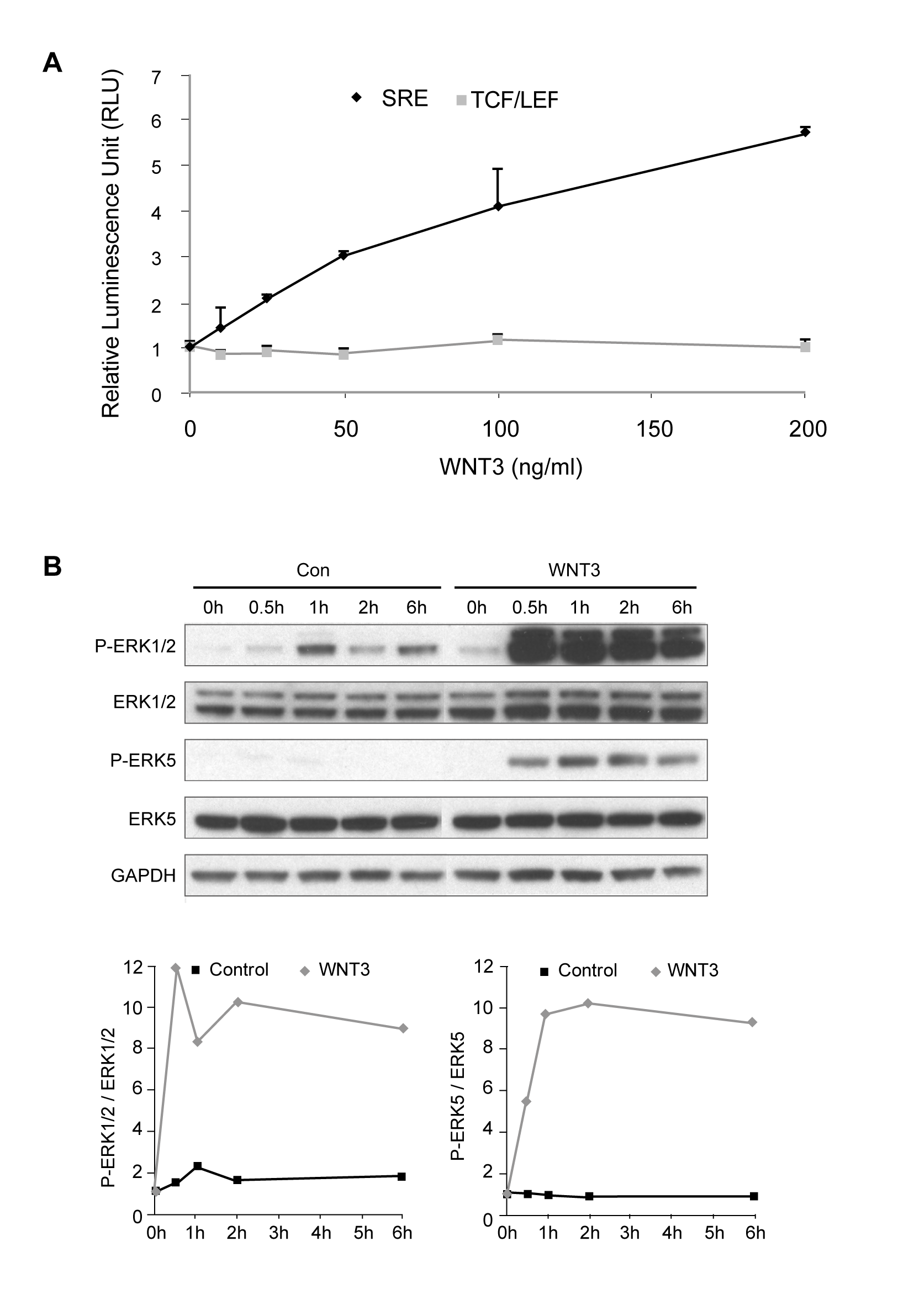

Supplement: Figure S3 — WNT3 regulation of MAPK signaling. (A) Using a Luciferase assay, MAPK signaling increases proportionally to WNT3 concentration in GCPs, which is reported by the expression of Firefly luciferase driven by the SRE promoter, but WNT3 fails to activate β-catenin signaling, which is reported by the expression of pTOPflash luciferase driven by the TCF/LEF promoter. (B) Activation of ERK1/2 and ERK5 in response to WNT3 treatment in GCPs. ERK phosphorylation is rapidly induced with WNT3 treatment. GCPs were treated with WNT3 for 0 to 6 h, and lysates were analyzed by Western blot analysis, using anti-P-ERK1/2, ERK1/2, P-ERK5 and ERK5 antibodies. GAPDH was used as a loading control. (Bottom panels) Quantification of P-ERK1/2 and P-ERK5 intensity normalized to ERK1/2 and ERK5 signal, respectively, as a function of time. (TIF) [file pone.0081769.s003.tif]

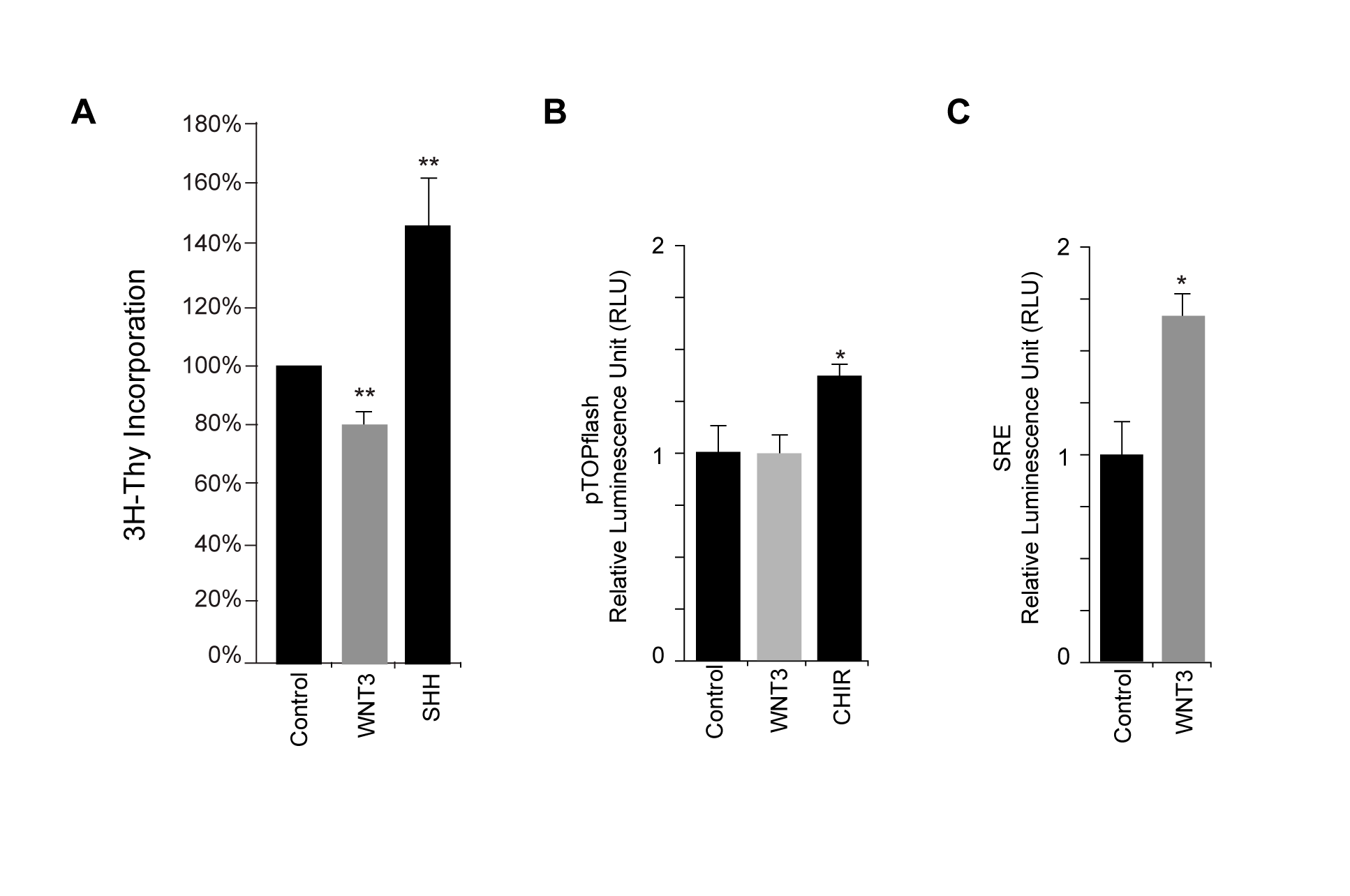

Supplement: Figure S4 — Commercially available, recombinant WNT3 inhibits proliferation in GCPs and activates MAPK signaling. (A) Commercially available WNT3 decreases GCP proliferation by 18.6 ± 3.51% compared to the control (n=4) as measured by [3H]-Thymidine incorporation assay. Statistical significance of WNT3 or SHH is compared to control. (B) Using a luciferase assay, commercially available WNT3 fails to activate β-catenin signaling, which is reported by the expression of pTOPflash luciferase driven by the TCF/LEF promoter. CHIR99021 (136.53% ± 5.23% of control, n=3) is an inhibitor of glycogen synthase kinase 3β (GSK3β) that activates canonical WNT/β-catenin signaling, and was used as a positive control to demonstrate that the canonical WNT signaling pathway is functional in cultured GCPs. GCPs were treated with 250 ng/ml of commercially available WNT3 (Creative Biomart) or 3 µM CHIR99021 (StemGent). (C) Test of commercially available WNT3 specificity using an SRE luciferase assay in GCPs. Commercially available WNT3 (167.04 ± 10.04% of control, n=3) activates MAPK signaling, reported by the expression of Firefly luciferase driven by a minimal MAPK promoter plus multiple SRE binding sites. Data represent the mean ± s.e.m.: *p<0.05, **p<0.01, ***p<0.001. NS, not significant. (TIF) [file pone.0081769.s004.tif]

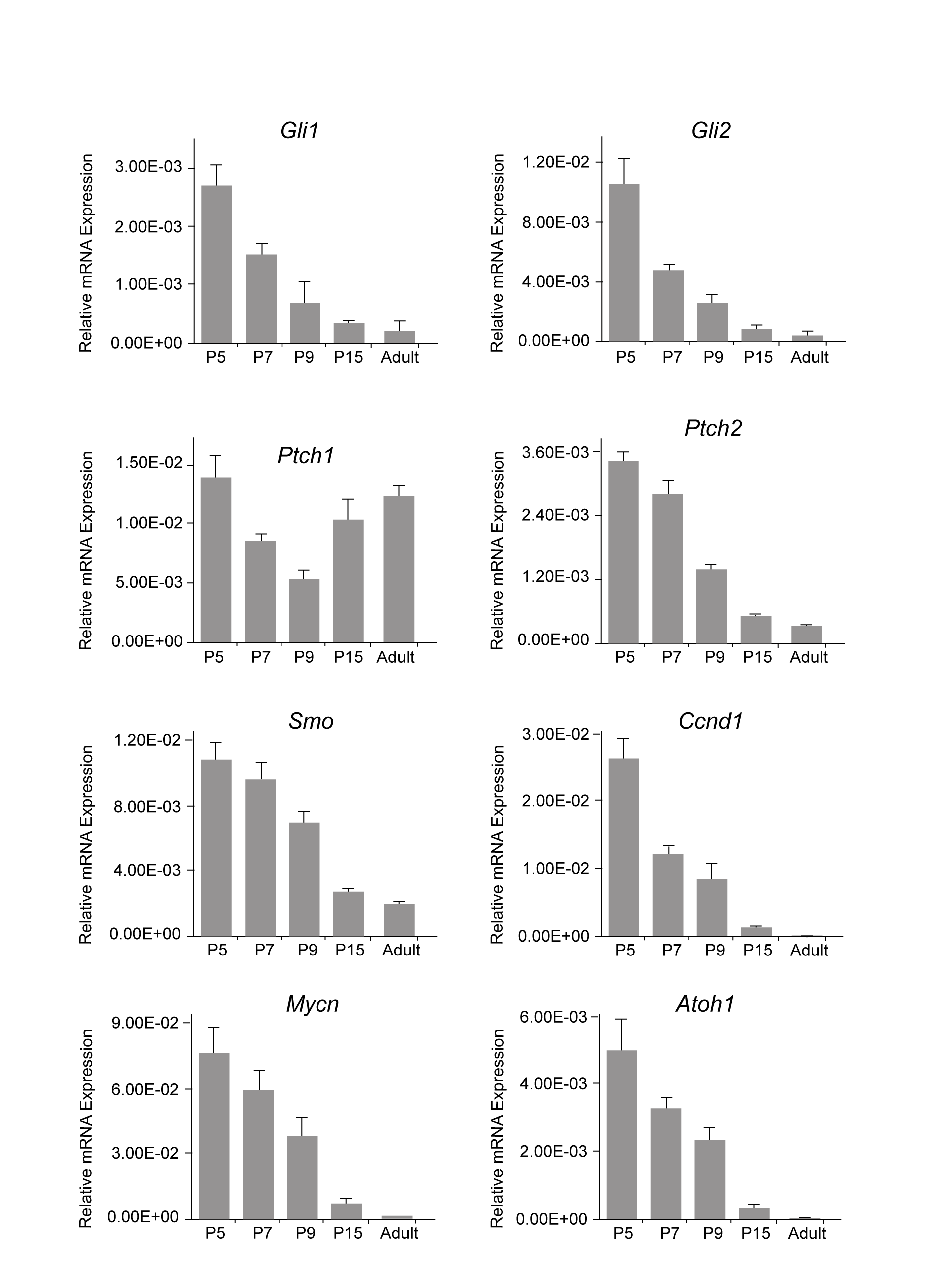

Supplement: Figure S5 — qPCR of SHH signaling components, including Gli1, Gli2, Ptch1, Ptch2, Smo, Ccnd1, Mycn, and a transcription factor expressed in proliferating GCPs, Atoh1, in the mouse cerebellum from P5 to adult. SHH signaling molecules and Atoh1 levels were normalized against β-2-microglobulin (B2M), hyporanthine-guanine phosphoribosyltransferase (HPRT1) and ribosomal subunit 18s (M18s). (TIF) [file pone.0081769.s005.tif]
