# Supplementary material for: WNT3 Inhibits Cerebellar Granule Neuron Progenitor Proliferation and Medulloblastoma Formation via MAPK Activation
Source: PLoS One. 2013 Nov 26;8(11):e81769. doi: 10.1371/journal.pone.0081769 (PMC3841149; doi:10.1371/journal.pone.0081769)
Supplement: Table S1 — List of qPCR primers. (DOCX) [file pone.0081769.s006.docx]

**Table 1**

| **qPCR primers** |  |
| --- | --- |
| *Wnt1*_Sense | ctactggcactgaccgctct |
| *Wnt1*_Antisense | ggaggctatgttcacgatgc |
| *Wnt3*_Sense | cacaacacgaggacggaga |
| *Wnt3*_Antisense | aatctaccccttcccagtgc |
| *Wnt3a*_Sense | agtgccagcaccagttcc |
| *Wnt3a*_Antisense | catggacaaaggctgactcc |
| *Wnt4*_Sense | ccgggcactcatgaatct |
| *Wnt4*_Antisense | cacgccagcacgtctttac |
| *Wnt5a*_Sense | tgaagcaggccgtaggac |
| *Wnt5a*_Antisense | agccagcacgtcttgagg |
| *Wnt5b*_Sense | gagagcgtgagaagaactttgc |
| *Wnt5b*_Antisense | ggcgacatcagccatcttat |
| *Wnt6*_Sense | tgtcagttccagttccgtttc |
| *Wnt6*_Antisense | agctgtctctcggatgtcct |
| *Wnt7a*_Sense | ggactatgacccggaaagc |
| *Wnt7a*_Antisense | cagagctaccaccgaagagaa |
| *Wnt8b*_Sense | ccagccatggtggacttc |
| *Wnt8b*_Antisense | cgaggctgcagtttctagtca |
| *Wnt10b*_Sense | ttcacgagtgtcagcacca |
| *Wnt10b*_Antisense | aaagcactctcacggaaacc |
| *Smo_Sense* | TTGTGCTCATCACCTTCAGC |
| *Smo_Antisense* | CAGGAATGGGCTTCTTGGTA |
| *Gli1_Sense* | CCTGGTGGCTTTCATCAACT |
| *Gli1_Antisense* | GTGGTACACAGGGCTGGACT |
| *Gli2_Sense* | ACCATGCCTACCCAACTCAG |
| *Gli2_Antisense* | CCTCAGCCTCAGTCTTGACC |
| *Atoh1_Sense* | ACATCTCCCAGATCCCACAG |
| *Atoh1_Antisense* | ACAACGATCACCACAGACCA |
| *Mycn_Sense* | GCGGTAACCACTTTCACGAT |
| *Mycn_Antisense* | AGTTGTGCTGCTGATGGATG |
| *Ki67_Sense* | CAGTACTCGGAATGCAGCAA |
| *Ki67_Antisense* | CAGTCTTCAGGGGCTCTGTC |
| *Notch2_Sense* | CCTGAACGGGCAGTACATTT |
| *Notch2_Antisense* | GCGTAGCCCTTCAGACACTC |
| *Zic2_Sense* | AAATATGAGCCGTGCCAAAG |
| *Zic2_Antisense* | AACGGCACAACGTTTACTCC |
| *Gabra6_Sense* | CGTTTTTCTGGCAAACCATT |
| *Gabra6_Antisense* | CGGTCACCCTCCTGTTTTTA |
| *Ptch1_Sense* | CTCAGGCAATACGAAGCACA |
| *Ptch1_Antisense* | GACAAGGAGCCAGAGTCCAG |
| *Ccnd1_Sense* | CAACAGGTTGTAGGGCTGGT |
| *Ccnd1_Antisense* | GGTAATGCCATCATGGTTCC |
| *Ptch2_Sense* | CTACATGGGGCTAACCGTGT |
| *Ptch2_Antisense* | TTTGTCGTGAAGCCA |
| *Hprt1_Sense* | GCCGAGGATTTGGAAAAAGTG |
| *Hprt1_Antisense* | GAACTTATAGCCCCCCTTGAGC |
| *M18s_Sense* | TTCGAACGTCTGCCCTATCAA |
| *M18s_Antisense* | ATGGTAGGCACGGCGACTA |
| *β 2M_Sense* | ATGGGAAGCCGAACATACT |
| *β 2M_Antisense* | CAGTCTCAGTGGGGGTGAAT |
| *β actin_Sense* | gccaaccgtgaaaagatgac |
| *β actin _Antisense* | gaggcatacagggacagcac |
| *Gapdh_Sense* | TGAACGGGAAGCTCACTGGCAT |
| *Gapdh_Antisense* | TCAGATGCCTGCTTCACCACCT |
